# Adhesion and Reconstruction of Graphene/Hexagonal Boron Nitride Heterostructures: A Quantum Monte Carlo Study

**DOI:** 10.1021/acsnano.4c10909

**Published:** 2025-02-10

**Authors:** Marcin Szyniszewski, Elaheh Mostaani, Angelika Knothe, Vladimir Enaldiev, Andrea C. Ferrari, Vladimir I. Fal’ko, Neil D. Drummond

**Affiliations:** † Department of Physics, Lancaster University, Lancaster LA1 4YB, U.K.; ‡ Department of Physics and Astronomy, University College London, London WC1E 6BT, U.K.; § Cambridge Graphene Centre, University of Cambridge, 9 J. J. Thomson Avenue, Cambridge CB3 0FA, U.K.; ∥ National Graphene Institute, University of Manchester, Booth Street East, Manchester M13 9PL, U.K.; ⊥ Institut für Theoretische Physik, Universität Regensburg, D-93040 Regensburg, Germany

**Keywords:** two-dimensional materials, graphene, hexagonal boron nitride, adhesion, quantum Monte Carlo

## Abstract

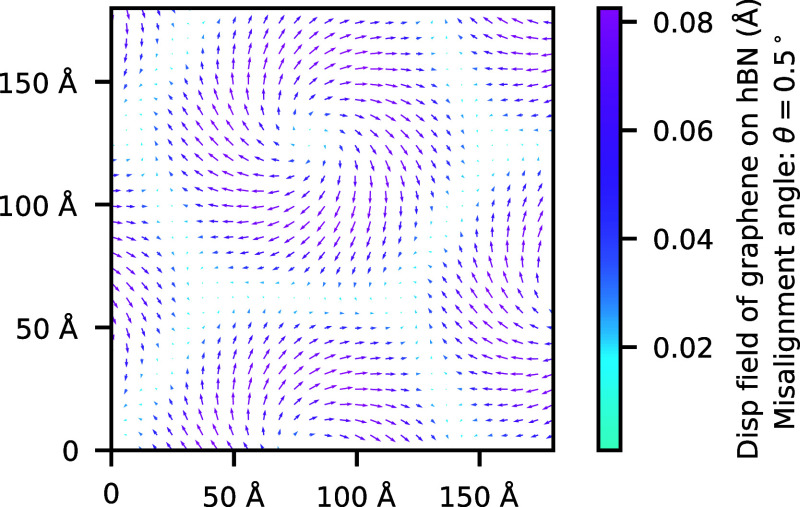

We investigate interlayer adhesion and relaxation at interfaces between graphene and hexagonal boron nitride (hBN) monolayers in van der Waals heterostructures. The adhesion potential between graphene and hBN is calculated as a function of local lattice offset using diffusion quantum Monte Carlo methods, which provide an accurate treatment of van der Waals interactions. Combining the adhesion potential with elasticity theory, we determined the relaxed structures of graphene and hBN layers at interfaces, finding no metastable structures. The adhesion potential is well described by simple Lennard–Jones pair potentials that we parametrize using our quantum Monte Carlo data. Encapsulation of graphene between near-aligned crystals of hBN gives rise to a moiré pattern whose period is determined by the misalignment angle between the hBN crystals superimposed over the moiré superlattice previously studied in graphene on an hBN substrate. We model minibands in such supermoiré superlattices and find them to be sensitive to the 180° rotation of one of the encapsulating hBN crystals. We find that monolayer and bilayer graphene placed on a bulk hBN substrate and bulk hBN/graphene/bulk hBN systems do not relax to adopt a common lattice constant. The energetic balance is much closer for free-standing monolayer graphene/hBN bilayers and hBN/graphene/hBN trilayers. The layers in an alternating stack of graphene and hBN are predicted to strain to adopt a common lattice constant, and hence, we obtain a stable three-dimensional crystal with a distinct electronic structure.

Layered material heterostructures (LMHs) comprise vertically stacked layered materials (LMs), held together by weak interlayer van der Waals (vdW) forces.^1−4^ For example, an LMH can be made by mechanically transferring monolayer graphene (1L-G) onto an atomically flat surface of a single-crystal hexagonal boron nitride (hBN) substrate.^5^ Monolayers (1L) of hBN have the same honeycomb structure as 1L-G, and they are insulators free of dangling bonds. Hence, they are one of the most preferred substrates for graphene (G) devices because they largely preserve 1L-G′s electronic properties.^5,6^ In 1L-G/1L-hBN heterostructures with near-aligned lattice vectors, quasiperiodic hexagonal moiré patterns^7−9^ with periods of up to 140 Å due to the small mismatch δ = 1 – *a*_G_/*a*_hBN_ ∼ 1.68% between the hexagonal lattice constants *a*_hBN_ = 2.504 Å of hBN^10^ and *a*_G_ = 2.462 Å of 1L-G^11,12^ lead to peculiar low-energy properties of electrons and holes. These moiré superlattice (SL) effects in 1L-G/1L-hBN are the result of an interplay between weak hybridization of electronic states near the Brillouin zone (BZ) corners in 1L-G with 1L-hBN orbitals and lattice relaxation of 1L-G, which deforms locally to reduce its adhesion potential to 1L-hBN.^9^

The encapsulation of G by bulk hBN (B-hBN) has become a widely used experimental technique. However, even when the lattice vectors of G and hBN are aligned, a diverse range of atomic configurations may arise in heterostructures of these materials, affecting their electronic properties. The impact of misalignment between layers on their electronic properties poses further challenges for experimental analysis. To address these issues and gain a deeper understanding of structural behavior at G/hBN interfaces, we have undertaken a study involving the combination of aligned 1L-G with 1L-hBN. Furthermore, our research contributes to overcoming the challenges in the field of graphene twistronics in hBN/G/hBN multilayered structures, an area of ongoing exploration in the scientific literature.^13−23^ In particular, we describe multiscale modeling of moiré SLs in 1L-G/B-hBN and 1L-G encapsulated between two aligned B-hBN crystals, describing the electronic structure of 1L-G in the resulting supermoiré pattern created by the top and bottom hBN in a B-hBN/1L-G/B-hBN LMH.

We have studied adhesion between 1L-G and 1L-hBN using the diffusion Monte Carlo (DMC) method^24,25^ as implemented in the casino code,^26^ previously used in studies of the binding properties of bilayer graphene (2L-G).^27^ We use the binding energies (BEs) *E*_bind_ (see eq 2 in the Methods section) computed for several 1L-G/1L-hBN stacking configurations, as shown in Table 1, and interlayer distances *d* to parametrize a symmetry-based interpolation formula describing  for an arbitrary *d* and in-plane offset  between the lattices of two crystallographically aligned 1Ls. We complete this description of local interlayer adhesion by using elasticity theory to describe the lattice relaxation of 1L-G adjusting to the underlying B-hBN lattice or to the lattices of two almost-aligned B-hBN crystals above and below the 1L-G.

**Table 1:**
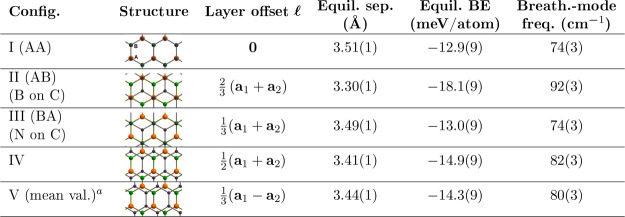
1L-G/1L-hBN Stacking Configurations and Corresponding Equilibrium Separations, BEs, and Breathing-Mode (Out-of-Plane Zone-Center Optical Phonon) Frequencies, Obtained by Fitting eq 1 to DMC Energy Data Obtained with Both Layers Forced to Adopt the Lattice Constant of G^b^,^c^,^d^,^e^,^f^,^g^

a See the Methods section for a detailed description of the configurations used.

b C, B, and N atoms are shown as black, orange, and green balls, respectively.

c Hexagonal sublattices A and B are labeled in the Config. I.

d Offset  is the in-plane displacement of each C–C bond center from the corresponding B–N bond center.

e **a**_1_ and **a**_2_ are the lattice vectors, as shown in Figure 1b.

f DMC equilibrium BEs of the different configurations are correlated due to the use of the same DMC 1L energies in each case; hence, the BE differences are more precise than suggested by the error bars on the absolute BEs.

g Errors on relative BEs are shown in Table 2.

Previous theoretical studies^8,28−31^ have addressed the interlayer adhesion in 1L-G/1L-hBN within the framework of density functional theory (DFT), using the local density approximation (LDA),^28^ or DFT-vdW methods combined with the random-phase approximation (RPA),^8,29−31^ which produced contradictory results for the BE of 2L-G.^32−36^

Here, we determine the BE and vibrational properties of a free-standing 1L-G/1L-hBN bilayer by using the DMC method. We performed calculations for 1L-G/1L-hBN rather than 1L-G on a B-hBN substrate because (i) the variation in BE of 1L-G on B-hBN substrate as a function of in-plane offset is dominated by the closest 1L-hBN to 1L-G^37^ and (ii) a 2L-LMH is much more computationally tractable than a bulk LMH for expensive quantum Monte Carlo methods, which scale as the third power of system size.^25^ G and hBN heterostructures are typically studied at low temperatures; hence, we performed all our calculations at zero temperature. Zero-point vibrational effects are neglected, since they largely cancel out the BE.

## Results and Discussion

### BE

**Figure 1:**
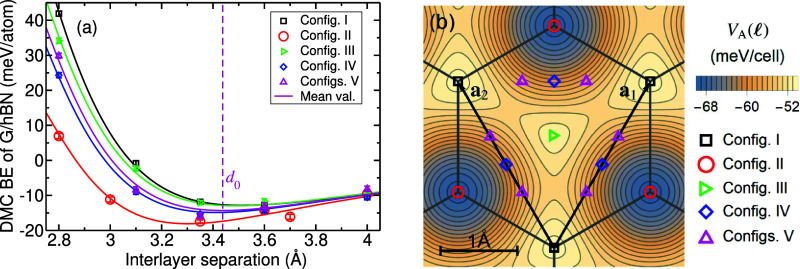
(a) DMC BE,  (eq 1), of 1L-G/1L-hBN as a function of interlayer separation *d* for the five stacking configurations in Table 1. Both layers have a lattice constant of 1L-G. Solid lines are a fit of eq 1 to the DMC data. Fitted parameters are reported in Table 1 of the Supporting Information (SI). (b) Adhesion potential per unit cell  as a function of the in-plane offset  of 1L-G relative to 1L-hBN at the layer separation *d*_0_ that minimizes the translationally averaged BE. Zigzag direction of the honeycomb lattice lies along the *x* axis. Even and odd parts of eq 1 for each of the stacking configurations are shown in Figure 1 of the SI.

The DMC-computed BE, , is plotted in Figure 1 against interlayer separation for 1L-G/1L-hBN in five different stacking configurations (i.e., five different offsets  of 1L-G relative to 1L-hBN, with both layers forced to adopt the 1L-G lattice constant). Because the elastic energy required to compress the 1L-hBN to have the same lattice constant as 1L-G cancels out of the BE,^38^ the precise choice of lattice constant does not affect the BE. The stacking configurations are labeled I–V and are shown in Table 1. Configuration I is AA stacking. Configurations II and III have AB (B on C) and BA (N on C) stacking. The BE curve of configuration II is flatter; therefore, we have studied a broader range of interlayer separations than for the other configurations. Configuration V is a “mean-value configuration,” for which the contribution to the offset-dependence of a quantity such as the BE from the first star of reciprocal-lattice vectors is zero. The DMC BEs per atom were extrapolated to the thermodynamic limit of infinite supercell size at a fixed primitive cell geometry. We fitted our DMC BEs by the function
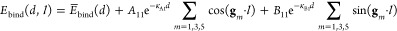
1where *E̅*_bind_(*d*) = *A*_01_*d*^–4^ + *A*_02_*d*^–8^ + *A*_03_*d*^–12^ + *A*_04_*d*^–16^, *d* is the interlayer separation, and  is the offset of the 1L-G lattice relative to the 1L-hBN lattice, with  corresponding to AA stacking (configuration I). For configuration V, . The {*A*_*sj*_}, {*B*_*sj*_}, {κ_A*s*_}, and {κ_B*s*_} are fitting parameters (see Table S1 of the SI for the fitted values), while **g**_*m*_ denotes the *m*th reciprocal-lattice point of the primitive cell and subscript *s* denotes the star of reciprocal-lattice vectors. We adopt the convention that reciprocal-lattice points are ordered by star *s*, starting with **g**_0_ = **0**, and that within each star successive reciprocal lattice points are labeled anticlockwise starting from the *y*-axis. The labeling is illustrated in Figure 4c of the SI. Thus, **g**_1_, **g**_3_, and **g**_5_ denote the first, third, and fifth reciprocal-lattice points in the first star of the hexagonal reciprocal lattice. eq 1 exhibits the expected^39^ configuration-independent *d*^–4^ vdW behavior at separation larger than a few times 1/min{κ_A1_, κ_B1_}, and satisfies the translational and *D*_3_ point symmetry in the offset . The minimum of *E̅*_bind_(*d*) in eq 1 provides the nominal equilibrium separation *d*_0_ for a rigid pair of misaligned LMs (because all offsets  are present in the moiré supercell), while the second derivative *E̅*_bind_″(*d*_0_) determines their layer breathing mode (LBM) frequency ω_BM_. The translationally averaged DMC breathing-mode frequency is 80(3) cm^–1^, in good agreement with the DFT-LDA result (∼77 cm^–1^; see Figure 2 of the SI). The reduced χ^2^ does not change significantly when two stars of nonzero reciprocal lattice vectors are used instead of one in the fitting function for the BE. Hence, for simplicity, we used the one-star model in eq 1.

Our DMC results show that stacking configuration II has the lowest energy, in agreement with DFT calculations.^28^ In this structure, the C 2p_*z*_ electrons are closer to the partially positive B atoms than to the partially negative N atoms.

Table 3 compares our DMC BEs with DFT for each stacking configuration. DFT-LDA underestimates the BE by up to 40%, while DFT-vdW overbinds 1L-G/1L-hBN by up to 50%. DFT-RPA^8^ overbinds 1L-G/1L-hBN by 25% compared with DMC. Relative BEs for the different stacking configurations are given in Table 2. Stacking configuration II is 5.2(4) meV/atom more stable than the least stable stacking configuration (I). This is similar to the difference of 5.25 meV/atom calculated by DFT-RPA^8^ and DFT-vdW.^40^ The difference in the DMC BEs of configurations I and III is negligible, as is also predicted by DFT-vdW^40^ and DFT-RPA.^8,29,30^ Even DFT-LDA correctly predicts the qualitative trend for the relative stability of the different stacking configurations. The DMC equilibrium separations of the different stacking configurations are in Table 1 and lie in the range 3.30–3.51 Å. This is similar to the range of separations ∼3.2 to 3.5 and ∼3.35 to 3.55 Å predicted by DFT-vdW^29,40^ and DFT-RPA^8^; see Table 2 of the SI.

**Table 2:** Differences in the Equilibrium BE of 1L-G/1L-hBN between the Different Stacking Configurations in Table 1 Calculated Using DFT and DMC^a^,^b^,^c^

	relative BE (meV/atom)			
difference	DFT-LDA	DFT-vdW^29,40^	DFT-RPA	DMC
*E*_I_ – *E*_II_	6, 6^28^ ∼9.5^29^	∼10, 5.25	5.25,^8^ 5^30^	5.2(4)
*E*_III_ – *E*_II_	5, ∼9.5^29^	∼10, 4.25	4.5,^8^ 4^30^	5.1(5)
*E*_IV_ – *E*_II_	3, 5,^28^ ∼4.5^29^	∼5, 3.0	4.5^8^	3.2(3)
*E*_V_ – *E*_II_	1			3.8(3)
*E*_I_ – *E*_III_	1, ∼0^29^	∼0, 1	0.75,^8^ 1^30^	0.1(4)

a *E*_*Y*_ is the equilibrium total energy per atom of 1L-G/1L-hBN in the stacking configuration *Y*.

b Both layers were assumed to have the 1L-G lattice constant in the present work and in refs (28, 29), while the lattice constant of 1L-hBN was used in refs (8, 40).

c Averaged lattice constant was used in ref (30) and the other DFT-LDA results are from the present work.

### Reconstruction of Perfectly Aligned G/hBN Composites into Lattice-Matched Structures

The total energy of 1L-G/1L-hBN has two relevant contributions: (i) adhesion potential *U*_A_ and (ii) elastic energy *U*_E_ due to the straining of the layers. Our DMC calculations show that, for a 1L-G/1L-hBN LMH in which both layers have the same lattice constant, the equilibrium BE is higher by 3.8(3) meV/atom in the mean-value configuration (V) than in the most stable stacking configuration (II); see Table 2. Hence, the adhesion potential could be lowered by 15(1) meV per 1L-G unit cell if 1L-G were to adopt the same lattice constant as hBN, forming stacking configuration II uniformly instead of a moiré pattern. Where 1L-G is encapsulated between two perfectly aligned regions of B-hBN, the adhesion potential could be lowered by as much as 30(2) meV per 1L-G unit cell if 1L-G adopts the hBN lattice constant.

**Table 3:** Equilibrium BE of 1L-G/1L-hBN from DFT^a^,^b^

	BE (meV/atom)		
Config.	DFT-LDA	DFT-vdW^29,40^	DFT-RPA
I	–9, ∼−10,^28^ ∼−7.5^29^	∼−25, −28.50	–15.5,^8^ −18^30^
II	–15, ∼−16,^28^ ∼−17^29^	∼−35, −33.75	–20.75,^8^ −23^30^
III	–10, ∼−7.5^29^	∼−25, −29.50	–16.25,^8^ −19^30^
IV	–12, ∼−11,^28^ ∼−12.5^29^	∼−30	–17.75^8^
V	–11		

a Both layers were assumed to have the 1L-G lattice constant in the present work and in refs (28, 29), while the 1L-hBN lattice constant was used in refs (8, 40).

b Averaged lattice constant was used in ref (30) and the other DFT-LDA results are from the present work.

The elastic energy  required to strain 1L-G to have lattice constant *a* may be calculated using eq 5 of the SI with strain tensor ε = (*a*/*a*_G_ – 1)*I*, where *I* is the 2 × 2 identity matrix. This gives , where λ_G_ and μ_G_ are the Lamé coefficients of 1L-G, whose experimentally measured values are given in the SI. Hence the elastic energy required to strain 1L-G to have the same lattice constant as hBN is  meV per 1L-G unit cell. Thus, the reduction in adhesion potential from adopting stacking configuration II uniformly does not compensate for the increase in the elastic energy, so the layers retain their different, incommensurate lattice constants, even when 1L-G is encapsulated in B-hBN and the crystallographic directions of the lattices are aligned. This, however, does not exclude local deformations, which adjust the two lattices periodically following the moiré pattern of the two slightly incommensurate crystals, leading to the weak lattice reconstruction that we describe in the SI. In these weak reconstructions, we do not find any evidence of nonglobal energy minima, implying that there are no low-energy metastable structures at 1L-G/1L-hBN interfaces.

For a suspended 1L-G/1L-hBN bilayer, the adhesion potential reduction  meV per unit cell due to adopting the most stable stacking configuration uniformly must again be compared with the change  in the total elastic energy.  is the sum of the elastic energies required to stretch/compress the individual layers to have the common lattice constant *a* such that the elastic energy is minimal with respect to *a*, i.e.,  meV per unit cell, where the expression for  is analogous to that given above for 1L-G. The elastic energy penalty is 4(1) meV per unit cell larger than the reduction in the adhesion potential, so that the layers retain their incommensurate lattice constants (just). For a suspended 1L-hBN/1L-G/1L-hBN trilayer with aligned 1L-hBN lattices, the adhesion potential reduction  meV per unit cell due to adopting a common lattice constant must be compared with the elastic energy penalty  meV per unit cell. Hence, the adhesion energy gain marginally exceeds the elastic energy penalty by 4(2) meV per unit cell so that the trilayer is expected to reconstruct to form a new 2d crystal. However, small strains may easily change the balance of the energies in these two free-standing structures and, hence, change the fate of lattice reconstruction. Finally, for a multilayer structure consisting of an alternating series of 1L-G and 1L-hBN layers,  meV per unit cell per 1L-G/1L-hBN bilayer and  meV per unit cell per 1L-G/1L-hBN bilayer, so the balance shifts completely in favor of adhesion, leading to a reconstructed lattice constant of *a′* = 2.481 Å and to a peculiar semimetallic band structure of this bulk composite material.^22^

Using the fitted adhesion potential, we have parametrized a continuum model of the local relaxation of 1L-G on a rigid hBN substrate (see Methods), allowing us to examine the electronic properties of B-hBN/1L-G/B-hBN^13−15,17−20,23^ for various orientations of the hBN unit cells (see SI).

### Effective Interatomic Pair Potentials Beyond Standard Lennard–Jones Theory

**Figure 2:**
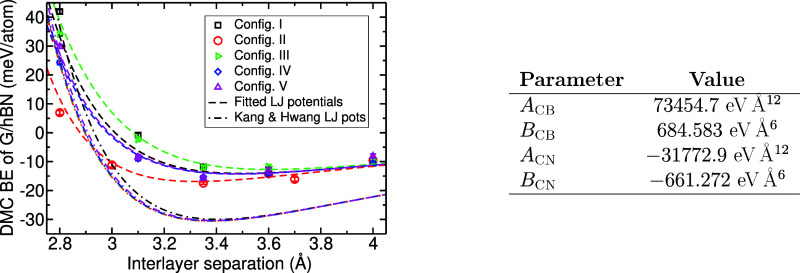
DMC 1L-G/1L-hBN BEs (markers) together with BE curves obtained using both our fitted LJ pair potentials (dashed lines) and those from ref (47). (dash-dotted lines). Both layers are constrained to have the 1L-G lattice constant. Our fitting parameters are reported in the right-hand panel. Equilibrium layer separations from our fitted pair potentials are 3.50, 3.32, 3.60, 3.48, and 3.48 Å for stacking configurations I–V, respectively, and the corresponding equilibrium BEs are −14.1, – 16.9, – 12.7, – 14.3, and −14.2 meV/atom. These may be compared with the results in Table 1, which were obtained by fitting eq 1 to the DMC data.

Molecular dynamics simulations with interatomic pair potentials are often used to model interactions between 1L-G and 1L-hBN. The simplest useful pair potential is the two-parameter Lennard–Jones (LJ) form,^41^*U*(*r*) = *A*/*r*^12^ – *B*/*r*^6^, used to study 1L-G on 1L-hBN^42−45^ and 6L-hBN.^46^ However, the accuracy of the LJ potentials in 1L-G/1L-hBN LMHs is questionable, e.g., the parameters *A*_CB_ = 32679 eV Å^12^ and *B*_CB_ = 20.748 eV Å^6^ for a C–B LJ potential and *A*_CN_ = 34545 eV Å^12^ and *B*_CN_ = 23.709 eV Å^6^ for a C–N LJ potential were obtained in ref (47) and used in refs (42, 45) to study 1L-G/1L-hBN interfaces. Interlayer BE curves for a 1L-G/1L-hBN LMH obtained with these LJ potentials are shown in Figure 2. These LJ potentials overbind 1L-G/1L-hBN by around 50% and fail to distinguish the different stacking configurations.

We determine the parameters *A*_CB_ and *B*_CB_ for LJ C–B potentials and *A*_CN_ and *B*_CN_ for C–N potentials by fitting the LJ BE to our DMC BE results for all of the stacking configurations in Figure 1. The fitted parameters and resulting LJ BE curves are plotted in Figure 2. The fitted parameters take counterintuitive values; e.g., in the C–N pair potential, the LJ parameters are negative so that the *r*^–12^ term is attractive and the *r*^–6^ term is repulsive. Hence, over the relevant range of interatomic distances, the C–B pair potential is attractive, while the C–N one is repulsive. Although standard LJ potentials with attractive *r*^–6^ tails give a poor description of the physics of interlayer interactions, our results demonstrate that interatomic pair potentials of the LJ form can provide a good description of 1L-G/1L-hBN adhesion provided one is willing to dispense with transferability.

## Conclusions

We performed DMC calculations of the binding of 1L-G and 1L-hBN, finding the BE for different stacking configurations (i.e., lattice offsets). We have used these BEs to argue that G and hBN retain different, incommensurate lattice constants for 1L-G on a B-hBN substrate, while G and hBN adopt a common lattice constant in a stacked heterostructure of alternating layers of 1L-G and 1L-hBN. Free-standing 1L-G/1L-hBN bilayers and 1L-hBN/1L-G/1L-hBN trilayers are close to the boundary between these two types of behavior.

As described in the SI, we have used our calculated adhesion potential to evaluate the relaxation of 1L-G on a B-hBN substrate and 1L-G encapsulated in B-hBN. This has allowed us to examine the electronic structure of B-hBN/1L-G/B-hBN, showing that the electronic structure depends on the relative orientation of the B-hBN lattices above and below the 1L-G.

Finally, we report pair potentials for modeling interactions between 1L-G and 1L-hBN.

## Methods

### Atomic Structure of 1L-G/1L-hBN at a Given Layer Separation

To determine the atomic structure of 1L-G/1L-hBN for a given layer separation, we pinned the lattice vectors, the mean interlayer distance, and the mean lattice offset, and we relaxed the remaining structural parameters, which describe the slight buckling of less than 0.003 Å of the layers, within DFT.^48^ We used ultrasoft pseudopotentials,^48,49^ a plane-wave cutoff energy of 25 Ha, a 15 × 15 Monkhorst-Pack k-point mesh, and Grimme’s dispersion-corrected^50^ Perdew–Burke Ernzerhof (PBE)^51^ functional in a periodic simulation cell of height 16 Å. We fixed the in-plane lattice constants of both 1L-G and 1L-hBN at the experimental 1L-G lattice constant *a*_G_ = 2.46 Å.^11,12^ The resulting bucklings are energetically insignificant. To calculate the BE of 1L-G/1L-hBN in Figure 1 for the Config. V, we have used similar configurations to the mean-value configuration (V), as shown in Table 1. For Config. V, we performed calculations in which the layer offsets at *d* = 2.8, 3.1, 3.35, 3.6, and 4 Å are , 0.08a_1_ – 0.04a_2_, 0.04a_1_ – 0.03a_2_, 0.34a_1_–0.33a_2_, and 1/3a_1_, respectively. Offsets corresponding to the relaxed structures were used in the fit of eq 1.

### Details of the Quantum Monte Carlo Approach

The BE per atom of 1L-G/1L-hBN can be written as
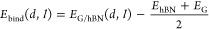
2where *E*_G/hBN_, *E*_hBN_, and *E*_G_ are the total energies per atom of 1L-G/1L-hBN, 1L-hBN, and 1L-G, calculated using the fixed-node DMC method as implemented in the casino code.^26^ We used Dirac–Fock pseudopotentials^52,53^ to represent the atomic cores, using the pseudopotential locality approximation.^54^ Our many-body trial wave functions consisted of Slater determinants for spin-up and spin-down electrons, multiplied by a Jastrow correlation factor^25^ containing polynomial and plane-wave electron–electron terms and polynomial electron–nucleus and electron–electron–nucleus terms.^55^ The Slater determinants contained Kohn–Sham orbitals^56^ generated using the castep plane-wave DFT code^48^ and re-expressed in a blip (B-spline) basis.^57^ Free parameters in our Jastrow factors were optimized within variational quantum Monte Carlo by unreweighted variance minimization.^58,59^ We generated the Kohn–Sham orbitals using the LDA functional and a plane-wave energy cutoff of 110 Ha. Although the DFT calculations were performed in a three-dimensionally periodic cell, the DMC calculations were carried out in a 2D periodic simulation supercell using a 2D Ewald interaction between charges.^60,61^

To remove biases due to finite time steps and populations of walkers, we performed pairs of DMC calculations using time steps in the ratio 1:2.5 with the corresponding target walker populations in the ratio 2.5:1, and we linearly extrapolated the resulting DMC energies to zero time step and infinite population. Time-step errors are discussed in the SI. The fixed-node error is of uncertain magnitude but is always positive.^62^ The regions of high electron density close to the nuclei make the dominant contribution to the fixed-node error, so fixed-node errors are expected to cancel largely when the BE is calculated.

To reduce quasirandom single-particle finite-size errors caused by momentum quantization, we evaluated twist-averaged^63^ DMC ground-state energies per atom in simulation supercells containing 3 × 3 and 5 × 5 unit cells for 1L-G, 1L-hBN, and 1L-G/1L-hBN. To remove systematic finite-size errors due to the long-range of the Coulomb interaction and two-body correlations, we extrapolated the twist-averaged energies to the thermodynamic limit of infinite system size. Twist averaging was performed by fitting
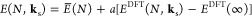
3to our DMC energies per atom *E*(*N*, *k*_s_) at twists (simulation-cell Bloch vectors) *k*_s_ in *N*-electron supercells, where *E̅*(*N*) and *a* are fitting parameters, *E*^DFT^(*∞*) is the DFT energy per atom obtained with a fine 50 × 50 *k*-point sampling and *E*^DFT^(*N*, *k*_s_) is the DFT energy with a *k*-point set corresponding to the DMC calculation. To maximize the cancelation of errors in the BE, at each system size, the same set of 12 random twists was used for the 1Ls and for 1L-G/1L-hBN at all separations and with all stacking configurations. The twist-averaged energies per atom *E̅*(*N*) were extrapolated to the thermodynamic limit of infinite system size by fitting
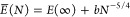
4to our data, where the energy per atom in the thermodynamic limit *E*(*∞*) and *b* are fitting parameters.^64^

### Continuum Model of Relaxation of Layers

To model the relaxation and deformation of 1L-G on a B-hBN substrate, we use a continuum model of the displacement field in an elastic 1L-G layer on rigid B-hBN.^38^ The misalignment angle between the lattice vectors of the elastic layer (1L-G) and the rigid layer (B-hBN) is denoted by θ and is assumed to be small. Let the unit cell positions of the elastic layer after relaxation be *r*_*n*_ + *u*(r_*n*_), where {*r*_*n*_} is the unrelaxed lattice points and *u*(*r*) is the displacement field. The displacement field is assumed to have the periodicity of the moiré supercell. We can therefore write the Fourier expansion of the displacement field as *u*(*r*) = ∑_*q*_*u*_q_e^*i* *q*·*r*^, where the sum runs over the reciprocal lattice points q of the moiré supercell.

In general, the adhesion potential per unit cell depends on θ. However, this dependence is negligible at small angles since the aligned case (θ = 0) is extremal. Hence, at small θ, the adhesion potential at any point in the bilayer simply depends on the local interlayer lattice offset . The spatial variation in the interlayer distance is small, so we evaluate the local contribution to the adhesion potential per unit cell as . Plots of the adhesion potential as a function of the offset of 1L-G relative to B-hBN are in Figure 1 and the SI (Figure 3). The expression for the adhesion potential is given in the SI.

In the continuum model, we write the total adhesion potential and the elastic energy in terms of the Fourier components of the displacement field. The equilibrium displacement field is then found by minimizing the sum of the elastic energy and total adhesion potential with respect to the Fourier components of the displacement field. Full details are given in the SI. Also given in the SI is an analysis of the consequences of lattice relaxation for the electronic structure of 1L-G.

## Data Availability

The data underlying this study are openly available at 10.17635/lancaster/researchdata/699.

## Supporting Information Available

BE Fitting Parameters; equilibrium separation, BE, and LBM frequency; DFT geometries; DFT phonon calculations; Lamé parameters; 2d adhesion potential parameters; relaxation of 1L-G/B-hBN within the continuum model; minimizing the analytical approximation to the total energy within the continuum model; Brute-force minimization of the total energy within the continuum model; modeling Moiré SL minibands for electrons in B-hBN/1L-G/B-hBN; band-structure reconstruction of B-hBN/1L-G/B-hBN: derivation of the Hamiltonian of shortest period; and time-step errors in DMC calculations (PDF)
