# Supplementary material for: Adhesion and Reconstruction of Graphene/Hexagonal Boron Nitride Heterostructures: A Quantum Monte Carlo Study
Source: ACS Nano. 2025 Feb 10;19(6):6014–20. doi: 10.1021/acsnano.4c10909 (PMC11841029; doi:10.1021/acsnano.4c10909)
Supplement: Supplementary file 1 — nn4c10909_si_001.pdf [file nn4c10909_si_001.pdf]

Supporting Information for "Adhesion and Reconstruction of Graphene/Hexagonal Boron Nitride Heterostructures: A Quantum Monte Carlo Study"

Marcin Szyniszewski,[†,‡] Elaheh Mostaani,[†,¶] Angelika Knothe,[§,‖] Vladimir Enaldiev,[§] Andrea C. Ferrari,[¶] Vladimir I. Fal'ko,[§] and Neil D. Drummond[*,†]

†*Department of Physics, Lancaster University, Lancaster LA1 4YB, United Kingdom*

‡*Department of Physics and Astronomy, University College London, London WC1E 6BT, United Kingdom*

¶*Cambridge Graphene Centre, University of Cambridge, 9 J. J. Thomson Avenue, Cambridge CB3 0FA, United Kingdom*

§*National Graphene Institute, University of Manchester, Booth Street East, Manchester M13 9PL, United Kingdom*

‖*Institut für Theoretische Physik, Universität Regensburg, D-93040 Regensburg, Germany*

E-mail: n.drummond@lancaster.ac.uk

BE Fitting Parameters

The fitting parameters in Equation 1 of the main text are shown in Table 1, while the even and odd parts of the BE are shown in Figure 1.

Table 1: Fitting parameters in Equation 1 of the main text for the BE of 1L-G/1L-hBN.

Parameter	Value	
A_{01}	-1.604928	eV Å$^{\mathbf{4}}$ / atom
A_{02}	-490.4703	eV Å$^{\mathbf{8}}$ / atom
A_{03}	74950.57	eV Å$^{\mathbf{12}}$ / atom
A_{04}	-1975507	eV Å$^{\mathbf{16}}$ / atom
A_{11}	57.39082	eV / atom
B_{11}	-16.12002	eV / atom
$\kappa_{\rm A1}$	3.363638	Å$^{\mathbf{-1}}$
$\kappa_{\rm B1}$	2.860883	Å$^{\mathbf{-1}}$

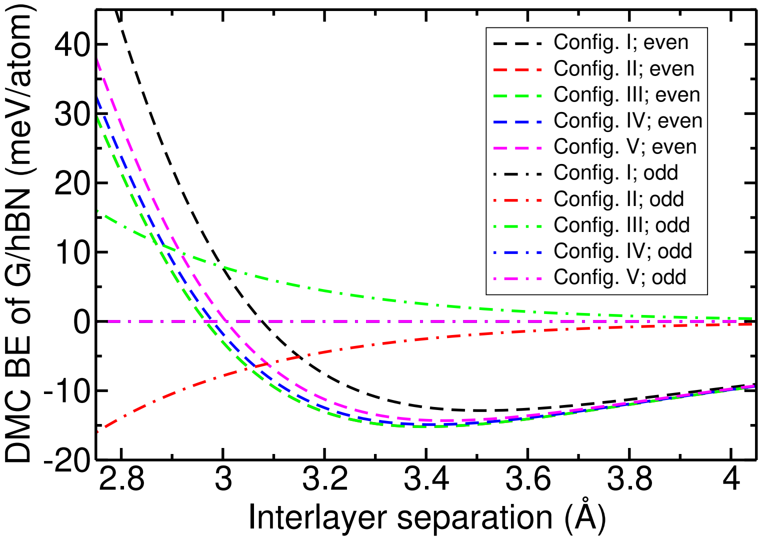

Figure 1: Even and odd parts of Equation 1 of the main text for each of the stacking configurations. The even BE parts of configurations II and III lie on top of each other, while the odd parts for configurations I, IV, and V are zero. The resultant BE curves are in Figure 1 of the main text.

Equilibrium Separation, BE, and LBM Frequency

The LBM frequency of an incommensurate 1L-G/1L-hBN bilayer is $\omega_{\rm BM} = 2\sqrt{\bar{E}''_{\rm bind}(d_0)/\mu}$, where d_0 is the equilibrium separation that minimizes the translationally averaged BE $\bar{E}_{\rm bind}(d)$ (the first term of Equation 1 of the main text), and $\mu = 2m_{\rm C}(m_{\rm B}+m_{\rm N})/(2m_{\rm C}+m_{\rm B}+m_{\rm N})$ is the reduced mass of a primitive cell of 1L-G/1L-hBN. $m_{\rm C}$, $m_{\rm B}$, $m_{\rm N}$ are the C, B, and N atomic masses. To evaluate the statistical error bars on d_0, $\bar{E}(d_0)$, and $\omega_{\rm BM}$ we used a bootstrap Monte Carlo procedure,[1] in which we repeatedly fitted Equation 1 of the main text to resampled DMC data drawn from Gaussian distributions centered on the mean DMC energy at each layer separation, with the standard deviation being the standard error in the mean energy at that layer separation. The mean and standard error in the mean of d_0, $\bar{E}(d_0)$, and $\omega_{\rm BM}$ are then accumulated.[2]

DFT Geometries

DFT-calculated interlayer distances for 1L-G/1L-hBN are reported in Table 2.

Table 2: Equilibrium interlayer separation of 1L-G/1L-hBN calculated by DFT. Both layers were assumed to have the 1L-G lattice constant in the present work and in Refs. 3 and 4, while the lattice constant of 1L-hBN was used in Refs. 5 and 6. An averaged lattice constant was used in Ref. 7. The other DFT-LDA results without citation are from the present work.

**Config.**	**Equilibrium interlayer separation (Å)**		
	**DFT-LDA**	**DFT-vdW**[4,6]	**DFT-RPA**
I	3.5, 3.50[3]	3.5, 3.49	3.55[5], 3.46[7]
II	3.2, 3.22[3]	3.2, 3.30	3.35[5], 3.27[7]
III	3.4, 3.40[3]	3.4	$\sim$ 3.4[5], 3.43[7]
IV	3.3	3.4, 3.45	3.5[5]
V	3.4		

DFT Phonon Calculations

DFT phonon dispersions were calculated using ultrasoft pseudopotentials and a plane-wave cutoff energy of 25 Ha within the LDA. We relaxed the lattice constant of the four-atom cell of 1L-G/1L-hBN. We used a 5×5 supercell with a 35×35 Monkhorst-Pack **k**-point mesh, and displaced the atoms by ± 0.04 Å to evaluate the matrix of force constants within the finite-displacement method. The initial equilibrium atomic positions were relaxed until the Hellmann-Feynman forces[8] were $< 5 \times 10^{-5}$ eV Å^{-1}.

Figure 2 plots the DFT-LDA phonon dispersion curves for 1L-G/1L-hBN in a four-atom cell at the relaxed common in-plane lattice constant 2.47 Å and the relaxed equilibrium separations of 3.5 and 3.2 Å for stacking configurations I and II, respectively. Since we are considering low frequencies close to acoustic branches, we do not calculate Kohn anomaly effects in 1L-G.

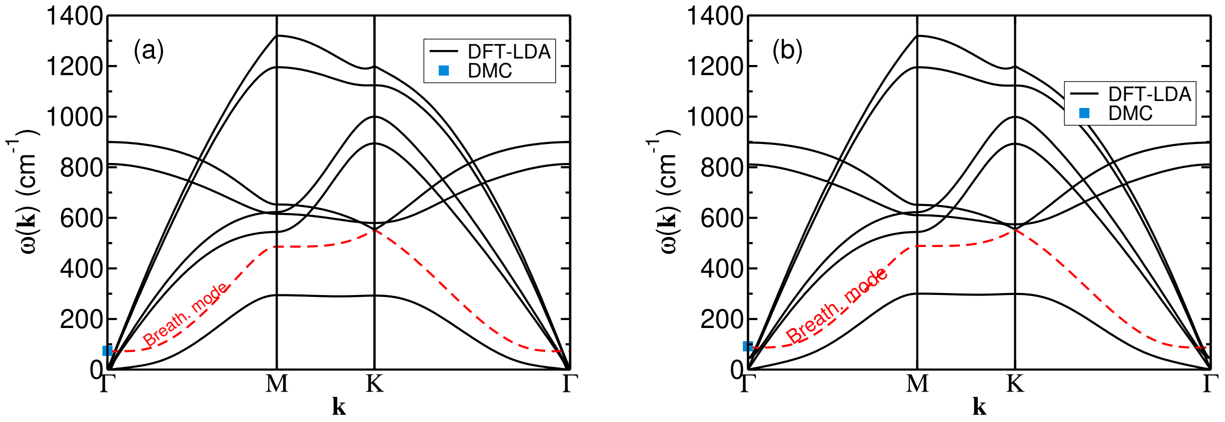

Figure 2: DFT-LDA phonon dispersions of 1L-G/1L-hBN for stacking configurations (a) I and (b) II. The branches that go to the LBM frequency at Γ are shown by dashed red curves and the DMC LBM frequencies at Γ point are shown by blue squares.

Lamé Parameters

The Young's modulus of bulk graphite is $Y = 1.02(3)$ TPa, and the in-plane Poisson's ratio is $\nu = 0.165$.[9] The lattice constants of Bernal-stacked graphite are $a = 2.461$ Å and $c = 6.708$

Å at 300 K.[10] The 2d Young's modulus of 1L-G is $Y_{2D} = Yc/2 = 340\ \mathrm{N\,m^{-1}} = 21.4\ \mathrm{eV\,Å^{-2}}$. The first Lamé parameter is $\lambda = Y_{2D}\nu/[(1+\nu)(1-2\nu)] = 4.5\ \mathrm{eV\,Å^{-2}}$. The second Lamé parameter is $\mu = Y_{2D}/[2(1+\nu)] = 9.2\ \mathrm{eV\,Å^{-2}}$. For thin graphene films, the Young's modulus was measured to be 1.0(1) TPa,[11] in agreement with the bulk graphite value.

The Lamé parameters of 1L-hBN are $\lambda_{\mathrm{hBN}} = 4.0\ \mathrm{eV\,Å^{-2}}$ and $\mu_{\mathrm{hBN}} = 7.4\ \mathrm{eV\,Å^{-2}}$, as determined by atomic force microscopy.[12]

2d Adhesion-Potential Parameters

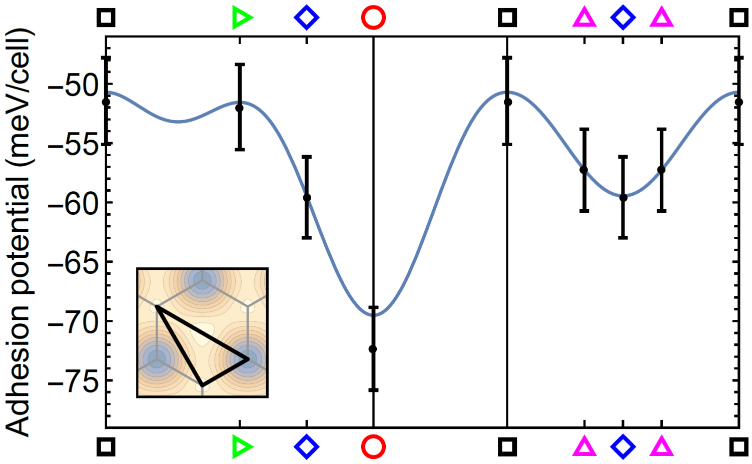

Figure 3: Adhesion potential V_A of 1L-G/B-hBN as a function of offset $\boldsymbol{\ell}$ of 1L-G relative to B-hBN around the triangular path through the unit cell shown in the inset. The DMC results are presented in black and $V_A(\boldsymbol{\ell})$ is in blue. The symbols indicate the stacking configurations in Table 1 and Figure 1 of the main text. Both layers have the 1L-G lattice constant. The error bars show the standard error in the mean of the BE at the minimum of the BE curve for each stacking configuration. The error bars on relative adhesion potentials are smaller than suggested by the error bars on the absolute values, due to the fact that the 1L energies cancel out of differences in adhesion potential.

To approximate the adhesion potential per unit cell $V_A(\boldsymbol{\ell})$ for 1L-G/B-hBN with in-plane offset $\boldsymbol{\ell}$ and a common lattice parameter, we use a truncated Fourier expansion:

$$\begin{aligned} V_A(\boldsymbol{\ell}) = \sum_m v_m e^{i\mathbf{g}_m\cdot\boldsymbol{\ell}} &\approx 4E_{\mathrm{bind}}(d_0,\boldsymbol{\ell}) \\ &\equiv v_{s0} + v_{s1}\sum_{m=1,3,5}\cos(\mathbf{g}_m\cdot\boldsymbol{\ell}) + v_{as1}\sum_{m=1,3,5}\sin(\mathbf{g}_m\cdot\boldsymbol{\ell}), \end{aligned} \quad (1)$$

where $\mathbf{g}_m$ is a reciprocal lattice point and E_{bind} is given in Equation 1 of the main text. From the fit of that equation, we find $v_{\text{s0}} = -57(3)$, $v_{\text{s1}} = 2.2(3)$, and $v_{\text{as1}} = -3.5(4)$ meV per primitive unit cell. The adhesion potential is plotted in Figure 3.

Relaxation of 1L-G/B-hBN within the Continuum Model

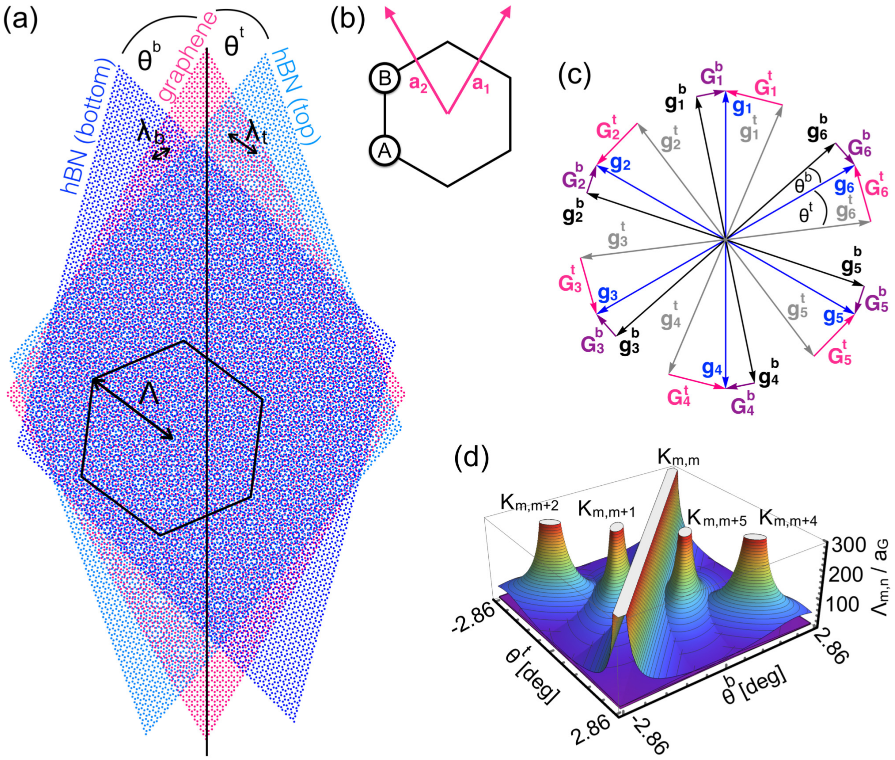

Figure 4: (a) Beating between the moiré patterns (of periods λ_{t} and λ_{b}) from the top and bottom 1L-G/B-hBN interfaces yields a supermoiré pattern of period Λ in the B-hBN/1L-G/B-hBN LMH. (b) Real-space lattice vectors of the 1L-G lattice. (c) Combining reciprocal lattice vectors to give the reciprocal lattice vectors $\mathbf{K}_{nm} = \mathbf{G}^{\text{t}}_n - \mathbf{G}^{\text{b}}_m$ of the supermoiré pattern. (d) Misalignment angles where the supermoiré reciprocal vectors $\mathbf{K}_{nm}$ vanish, and the corresponding supermoiré lattice vector $\mathbf{\Lambda}_{nm}$ diverges.

When we allow an elastic layer to relax on a rigid substrate, the adhesion potential V_{A} provides the energy of a unit cell at position $\mathbf{r}$, because $V_{\text{A}}(\mathbf{r})$ has the periodicity of the rigid lattice, and hence $V_{\text{A}}(\mathbf{r}) = V_{\text{A}}(\boldsymbol{\ell}_{\mathbf{r}})$, where $\boldsymbol{\ell}_{\mathbf{r}}$ is the position of the unit cell of the elastic layer relative to the closest rigid lattice point. In other words, $\boldsymbol{\ell}_{\mathbf{r}}$ is simply the local offset of the

elastic lattice relative to the rigid lattice. Let $\mathbf{g}_m$ and $\mathbf{g}_m^{\mathrm{b}}$ be the reciprocal lattice points of the unrelaxed elastic layer and the rigid ("bottom") layer, respectively. We may write the Fourier expansion of the adhesion potential as $V_{\mathrm{A}}(\boldsymbol{\ell}) = \sum_m v_m e^{i\mathbf{g}_m^{\mathrm{b}}\cdot\boldsymbol{\ell}}$ (see Equation 1). The difference of each corresponding pair of reciprocal-lattice points $\mathbf{g}_m$ and $\mathbf{g}_m^{\mathrm{b}}$ defines a reciprocal lattice point $\mathbf{G}_m^{\mathrm{b}} = \mathbf{g}_m - \mathbf{g}_m^{\mathrm{b}}$ of the moiré supercell; see Figure 4c. The total adhesion potential per unit cell of the elastic layer can be written as

$$\begin{aligned} U_{\mathrm{A}} = \frac{1}{N}\sum_{n=1}^{N} V_{\mathrm{A}}(\mathbf{r}_n + \mathbf{u}(\mathbf{r}_n)) &= \frac{1}{N}\sum_{n=1}^{N}\sum_m v_m e^{i\mathbf{g}_m^{\mathrm{b}}\cdot\mathbf{r}_n} e^{i\mathbf{g}_m^{\mathrm{b}}\cdot\mathbf{u}(\mathbf{r}_n)} \\ &= \frac{1}{N}\sum_{n=1}^{N}\sum_m v_m e^{-i\mathbf{G}_m^{\mathrm{b}}\cdot\mathbf{r}_n} e^{i\mathbf{g}_m^{\mathrm{b}}\cdot\mathbf{u}(\mathbf{r}_n)} \qquad (2) \\ &\approx v_0 + i\sum_m v_m \mathbf{g}_m^{\mathrm{b}} \cdot \mathbf{u}_{\mathbf{G}_m^{\mathrm{b}}}. \qquad (3) \end{aligned}$$

Equation 2 uses $e^{i\mathbf{g}_m^{\mathrm{b}}\cdot\mathbf{r}_n} = e^{i(\mathbf{g}_m^{\mathrm{b}} - \mathbf{g}_m)\cdot\mathbf{r}_n} = e^{-i\mathbf{G}_m^{\mathrm{b}}\cdot\mathbf{r}_n}$. Equation 3 assumes the displacement field to be small compared to the rigid lattice constant, which is the case if

$$|\mathbf{u}| \ll a^{\mathrm{b}}/(2\pi), \qquad (4)$$

where a^{b} is the lattice parameter of the rigid layer.

The elastic energy per unit cell is

$$\begin{aligned} U_{\mathrm{E}} &= \frac{1}{N}\int_{\text{crystal}} \frac{1}{2}\left[\lambda \mathrm{Tr}(\varepsilon)^2 + 2\mu \mathrm{Tr}(\varepsilon^2)\right] d^2\mathbf{r} \qquad (5) \\ &= \frac{1}{2}\sum_{\mathbf{q}} \mathbf{u}_{\mathbf{q}}^{\dagger} W_{\mathbf{q}} \mathbf{u}_{\mathbf{q}}, \qquad (6) \end{aligned}$$

where $\varepsilon_{ij} = (\partial u_i/\partial x_j + \partial u_j/\partial x_i)/2$ is the 2d pure strain field, λ and μ are the Lamé coefficients of the elastic layer, and N is the number of unit cells in the elastic layer. The dynamical matrix at wavevector $\mathbf{q}$ is[13]

$$W_{\mathbf{q}} = A\left[(\lambda + \mu)\mathbf{q}\mathbf{q}^{\mathrm{T}} + \mu q^2 I\right], \qquad (7)$$

where $A = |\mathbf{a}_1 \times \mathbf{a}_2|$ is the area of an unrelaxed primitive unit cell and I is the 2×2 identity matrix.

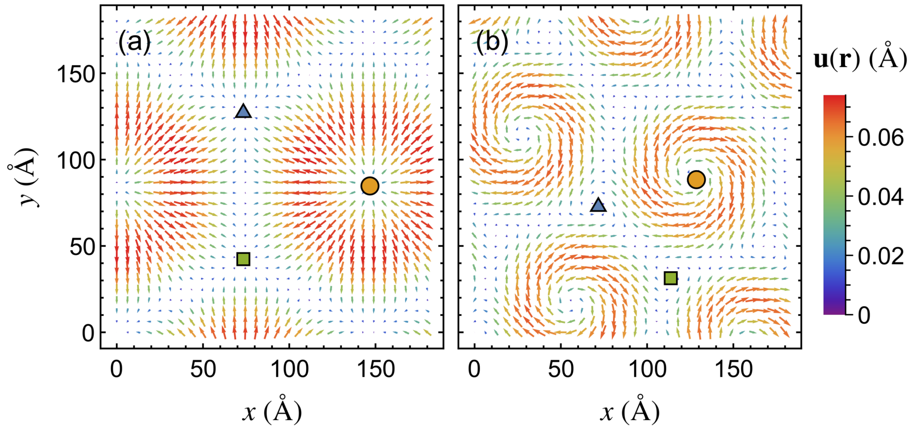

Figure 5: Displacement field $\mathbf{u}(\mathbf{r})$ of 1L-G unit cells in 1L-G/B-hBN LMHs, obtained by numerically minimizing the total energy of Equation 2 within the continuum model. The misalignment angle between the two layers is (a) $\theta = 0$; (b) $\theta = 1°$. The symbols indicate the stacking configurations shown in Table 1 of the main text.

By minimizing the total energy $U = U_{\mathrm{E}} + U_{\mathrm{A}}$ with respect to the Fourier components of the displacement field, we find the Fourier components of the displacement field (which has the periodicity of the moiré superlattice) to be

$$\mathbf{u}_{\mathbf{G}_m^{\mathrm{b}}} = i v_m^* W_{\mathbf{G}_m^{\mathrm{b}}}^{-1} \mathbf{g}_m^{\mathrm{b}}. \tag{8}$$

(See the section below for the derivation of Equation 8.) This result holds for any reciprocal lattice vector m. We do not assume any particular symmetry or structure in the derivation of Equation 8, so that Equation 8 is valid for any situation where one LM is allowed to relax after being transferred onto a substrate of another LM with a sufficiently similar lattice constant. Using a numerical evaluation of the right-hand side of Equation 2, we have investigated the continuum model without the approximation of small displacement fields. See the section below for details. For bilayers with greatly enhanced variations in the adhesion potential as a function of lattice offset or with greatly reduced elastic parameters, this method finds multiple energy minima. However, for 1L-G/B-hBN LMHs, we only find

the one, global minimum of the energy over the range of displacement fields ($\ll a_{\mathrm{hBN}}$) for which the continuum model applies. For aligned lattice vectors, this global minimum is 0.82 μeV per primitive cell lower than the energy predicted by the analytical approximation of Equation 8, which finds that the 1L-G relaxation lowers the energy by 0.37 meV per primitive cell. Physically, the stiffness of the two layers is too great to be overcome by the variations in the adhesion potential, which only weakly perturbs the 1L-G atomic structure. The displacement field that minimizes the energy is shown in Figure 5.

Minimizing the Analytical Approximation to the Total Energy within the Continuum Model

We assume finite-temperature effects to be negligible. Under zero external stress, the relaxed displacement field is that which minimizes the total energy. Under the assumption of a small displacement field the total energy can be written as:

$$U = U_{\mathrm{E}} + U_{\mathrm{A}} \approx \frac{1}{2}\sum_{\mathbf{q}} \mathbf{u}_{\mathbf{q}}^{\dagger} W_{\mathbf{q}} \mathbf{u}_{\mathbf{q}} + v_0 + i\sum_{m} v_m \mathbf{g}_m^{\mathrm{b}} \cdot \mathbf{u}_{\mathbf{G}_m^{\mathrm{b}}}, \tag{9}$$

as shown in Equation 3. We therefore require the derivatives of the energy with respect to the independent complex Fourier components of the displacement field to be zero, *i.e.*, $\nabla_{\mathbf{u}_{\mathbf{q}}} U = \mathbf{0}^{\mathrm{T}}$ and $\nabla_{\mathbf{u}_{\mathbf{q}}^{\dagger}} U = \mathbf{0}$. $W_{\mathbf{q}}$ is real and symmetric, and $W_{\mathbf{q}} = W_{-\mathbf{q}}$. Since $\mathbf{u}(\mathbf{r})$ is real, $\mathbf{u}_{\mathbf{q}}^{*} = \mathbf{u}_{-\mathbf{q}}$. So

$$\begin{aligned}
\nabla_{\mathbf{u}_{\mathbf{q}}} U &= \nabla_{\mathbf{u}_{\mathbf{q}}} \left(\frac{1}{2}\sum_{\mathbf{q}} \mathbf{u}_{\mathbf{q}}^{\dagger} W_{\mathbf{q}} \mathbf{u}_{\mathbf{q}} \right) + i\sum_{m} v_m (\mathbf{g}_m^{\mathrm{b}})^{\mathrm{T}} \delta_{\mathbf{q},\mathbf{G}_m^{\mathrm{b}}} \\
&= \nabla_{\mathbf{u}_{\mathbf{q}}} \left(\frac{1}{2}\mathbf{u}_{\mathbf{q}}^{\dagger} W_{\mathbf{q}} \mathbf{u}_{\mathbf{q}} + \frac{1}{2}\mathbf{u}_{-\mathbf{q}}^{\dagger} W_{-\mathbf{q}} \mathbf{u}_{-\mathbf{q}} \right) + i\sum_{m} v_m (\mathbf{g}_m^{\mathrm{b}})^{\mathrm{T}} \delta_{\mathbf{q},\mathbf{G}_m^{\mathrm{b}}} \\
&= \mathbf{u}_{\mathbf{q}}^{\dagger} W_{\mathbf{q}} + i\sum_{m} v_m (\mathbf{g}_m^{\mathrm{b}})^{\mathrm{T}} \delta_{\mathbf{q},\mathbf{G}_m^{\mathrm{b}}}
\end{aligned} \tag{10}$$

and, similarly,

$$\begin{aligned}
\nabla_{\mathbf{u}_\mathbf{q}^\dagger} U &= \nabla_{u_\mathbf{q}^\dagger}\left(\frac{1}{2}\mathbf{u}_\mathbf{q}^\dagger W_\mathbf{q}\mathbf{u}_\mathbf{q} + \frac{1}{2}\mathbf{u}_{-\mathbf{q}}^\dagger W_{-\mathbf{q}}\mathbf{u}_{-\mathbf{q}}\right) + i\nabla_{\mathbf{u}_\mathbf{q}^\dagger}\sum_m v_m^*(-\mathbf{g}_m^\mathrm{b})\cdot\mathbf{u}_{-\mathbf{G}_m^\mathrm{b}} \\
&= \nabla_{u_\mathbf{q}^\dagger}\left(\mathbf{u}_\mathbf{q}^\dagger W_\mathbf{q}\mathbf{u}_\mathbf{q}\right) + i\nabla_{\mathbf{u}_\mathbf{q}^\dagger}\sum_m -v_m^*\mathbf{u}_{\mathbf{G}_m^\mathrm{b}}^\dagger\mathbf{g}_m^\mathrm{b} \\
&= W_\mathbf{q}\mathbf{u}_\mathbf{q} - i\sum_m v_m^*\mathbf{g}_m^\mathrm{b}\delta_{\mathbf{q},\mathbf{G}_m^\mathrm{b}}. \qquad (11)
\end{aligned}$$

Therefore, the displacement field that minimizes the total energy of 1L-G/B-hBN satisfies

$$\mathbf{u}_\mathbf{q}^\dagger = -i\sum_m v_m(\mathbf{g}_m^\mathrm{b})^\mathrm{T}W_\mathbf{q}^{-1}\delta_{\mathbf{q},\mathbf{G}_m^\mathrm{b}} \quad \text{and} \quad \mathbf{u}_\mathbf{q} = i\sum_m v_m^* W_\mathbf{q}^{-1}\mathbf{g}_m^\mathrm{b}\delta_{\mathbf{q},\mathbf{G}_m^\mathrm{b}}, \qquad (12)$$

so that $\mathbf{u}_\mathbf{q}$ and $\mathbf{u}_\mathbf{q}^\dagger$ are an adjoint pair. This gives us Equation 8.

Brute-Force Minimization of the Total Energy within the Continuum Model

We wrote a program to evaluate the total energy per unit cell within the continuum model, Equation 2, by brute-force summation over 1L-G lattice sites, with the Fourier components of the displacement field determined by numerical minimization of the total energy using the conjugate-gradients method.[14] Initial real and imaginary parts of the Fourier components of the displacement field in the x- and y-directions are chosen randomly from a uniform distribution. The condition $\mathbf{u}(-\mathbf{q}) = \mathbf{u}^*(\mathbf{q})$ is imposed to ensure that the displacement field is real, but no other constraints are imposed on the Fourier coefficients.

For aligned 1L-G and B-hBN lattice vectors, the total energy was evaluated by summing over $N = 173889$ 1L-G unit cells, corresponding to a 7×7 array of moiré supercells. The Fourier expansion of the displacement field included moiré supercell reciprocal lattice vectors up to a magnitude of 1 Å^{-1}. This ensures that we can describe features on the scale of a single 1L-hBN unit cell (*i.e.*, the limit of validity of the continuum model). The procedure

was repeated for 240 different initial $\mathbf{u}_{\mathbf{G}_m^{\mathrm{b}}}$, randomly chosen with magnitudes up to 10 Å.

Modelling Moiré SL Minibands for Electrons in B-hBN/1L-G/B-hBN

For 1L-G encapsulated between two B-hBN crystals,[15–22] B-hBN/1L-G/B-hBN, the displacement field $\mathbf{u}^{\mathrm{t/b}} = \sum_m \mathbf{u}_{\mathbf{G}_m^{\mathrm{t/b}}} e^{-i\mathbf{G}_m^{\mathrm{t/b}}\cdot\mathbf{r}}$ can be approximated as a sum of the displacements arising from the top (t) B-hBN at twist angle θ^{t} and bottom (b) B-hBN at twist angle θ^{b}, each described using Equation 8. Such deformations lead to a complex moiré SL pattern experienced by the electrons in the 1L-G, which differs for B-hBN layers with parallel and antiparallel orientations of their unit cells (the latter case has $\theta^{\mathrm{t}} = \theta^{b} + 180°$).

A highly aligned 1L-G/B-hBN interface produces a perturbation $\delta\mathcal{H}$ to Dirac electrons, with Hamiltonian $\mathcal{H} = v_{\mathrm{F}}\mathbf{p}\cdot\boldsymbol{\sigma} + \delta\mathcal{H}$,[23] where $v_{\mathrm{F}} = 10^6\ \mathrm{m\,s^{-1}}$ is the 1L-G Fermi velocity,[24] $\mathbf{p}$ is the momentum operator and $\boldsymbol{\sigma}$ is the vector of Pauli matrices. In a B-hBN/1L-G/B-hBN system, the perturbations from the top (t) and bottom (b) interfaces superimpose and we obtain the combined perturbation,[25]

$$\delta\mathcal{H} = \delta\mathcal{H}^{\mathrm{t}} + \delta\mathcal{H}^{\mathrm{b}} = \sum_{\alpha=\mathrm{t,b}} \sum_{n=1}^{6} \left[U_0^{\alpha} + (-1)^n \left(iU_3^{\alpha}\sigma_3 + U_1^{\alpha}\mathbf{e}_n\cdot\boldsymbol{\sigma}\right)\right] e^{i\mathbf{G}_n^{\alpha}\cdot(\mathbf{r}\mp\mathbf{R}/2)}\, e^{i\mathbf{g}_n\cdot\mathbf{u}_{\mathbf{R}}(\mathbf{r})}, \quad (13)$$

where $\mathbf{e}_n$ are unit vectors in the directions of the 1L-G lattice points in the first star in real space, $\mathbf{R}$ is the reference shift between the top and bottom B-hBN crystals, and $\mathbf{u}_{\mathbf{R}}(\mathbf{r}) = \mathbf{u}^{\mathrm{t}}(\mathbf{r}-\mathbf{R}/2)+\mathbf{u}^{\mathrm{b}}(\mathbf{r}+\mathbf{R}/2)$. The parameters take into account the 1L-G displacement caused by the top and bottom B-hBN crystals. The parameters $U_0^{\mathrm{t/b}} = 16.4\frac{4\pi\sqrt{\delta^2+(\theta^{\mathrm{t/b}})^2}}{\sqrt{3}a_{\mathrm{G}}}$ eV Å, $U_1^{\mathrm{t/b}} = -32.8\frac{4\pi\delta}{\sqrt{3}a_{\mathrm{G}}}$ eV Å, and $U_3^{\mathrm{t/b}} = -32.8\frac{2\pi\sqrt{\delta^2+(\theta^{\mathrm{t/b}})^2}}{a_{\mathrm{G}}}$ eV Å are taken from the earlier moiré SL model in aligned 1L-G/B-hBN LMHs, and $\{\mathbf{G}_n^{t/b}\}$ are the first star of reciprocal lattice vectors of the SLs corresponding to the top/bottom interfaces.

We consider two mechanisms that give rise to supermoiré perturbations to the electronic

structure. Firstly, the interference between the top and bottom moiré patterns leads to electrons scattering off both lattices with respective reciprocal-lattice vectors $\mathbf{G}_m^{\mathrm{t/b}}$. This can be described within second-order perturbation theory in $\delta\mathcal{H}^{\mathrm{t/b}}(\mathbf{u}=\mathbf{0})$ as scattering with the combined reciprocal-lattice vectors $\mathbf{K}_{nm}=\mathbf{G}_n^{\mathrm{t}}-\mathbf{G}_m^{\mathrm{b}}$. Secondly, mixing between top and bottom moiré patterns occurs due to 1L-G lattice relaxation caused by the combined influences of the top and bottom B-hBN. Using the Fourier representation of the displacement field, we perform an expansion in Equation 13 for small displacement fields (Equation 4). The resulting expression features different pairs of the moiré reciprocal-lattice vectors $\mathbf{G}_m^{\mathrm{t/b}}$, which combine to give the effective reciprocal-lattice vectors $\mathbf{K}_{nm}$, each describing one of the emergent periodic supermoiré structures, *i.e.* different beats between moiré patterns at the top and bottom interfaces.

If one of the top or bottom B-hBN layers is strongly misaligned with respect to the 1L-G, while the other is almost aligned (*e.g.* $\theta^{\mathrm{t}}\gg\theta^{\mathrm{b}}\sim 0$), all the supermoiré reciprocal-lattice vectors are large, so that there is no effect of the supermoiré periodic structure at low energies. However, strain from the near-aligned layer opens a gap:

$$\Delta_{\mathrm{S}}(\theta^{\mathrm{b}})=-6U_3w_{\mathrm{as}}, \tag{14}$$

where $w_{\mathrm{as}}^{\mathrm{t/b}}=-(-1)^m\mathrm{Re}(\mathbf{g}_m\cdot\mathbf{u}_{\pm\mathbf{G}_m^{\mathrm{t/b}}})$. The gap is plotted against misalignment angle in Figure 6a.

For any supermoiré reciprocal-lattice vector $\mathbf{K}_{nm}$ the real-space period of the corresponding supermoiré pattern is $\Lambda_{nm}=\frac{4\pi}{\sqrt{3}}\frac{1}{K_{nm}}$. In the following we focus on the longest-period effects, and hence keep only the shortest nonzero $\mathbf{K}_{nm}$. As we illustrate in Figure 4d, for certain misalignment angles of the top and bottom B-hBN layers in B-hBN/1L-G/B-hBN, combinations of the form $\mathbf{K}_{nm}=\mathbf{G}_n^{\mathrm{t}}-\mathbf{G}_m^{\mathrm{b}}$ become very small and can even vanish completely. Hence these combinations yield the shortest reciprocal-lattice vectors at those misalignment angles. The most generic conditions for long-period beats are for $m=n$, for which the

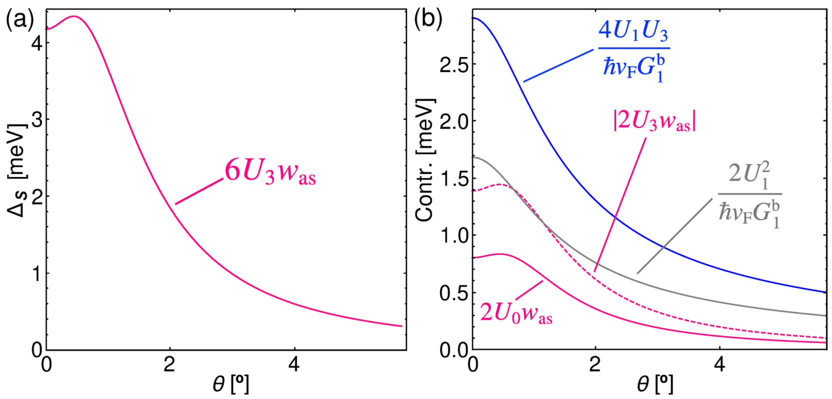

Figure 6: (a) Strain-induced energy gap Δ_S of a single interface between 1L-G and B-hBN in B-hBN/1L-G/B-hBN LMH (b) Comparison of the magnitudes of the different energy contributions in Equations 15 and 16.

period of the supermoiré lattice diverges for any stacking configuration with $\theta^t = \theta^b$. We now discuss the corresponding low-energy Hamiltonians and properties of this configuration for the relevant low-energy regime with $\theta^t \approx \theta^b$. Since 1L-hBN does not preserve inversion symmetry, the configurations in which the top and bottom B-hBN layers are parallel and antiparallel yield distinct cases.

The perturbative term in the electronic 1L-G Hamiltonian for the case in which the top and bottom B-hBN are aligned ($\theta^t = \theta^b$) is

$$\begin{aligned}\delta\mathcal{H}_{\rm par} =& -12U_3 w_{\rm as}\sigma_3 \\ & - \sum_{n=1}^{6}\left[2U_3 w_{\rm as}\sigma_3 + \frac{4U_1U_3}{\hbar v_{\rm F} G_n^{\rm b}} + i\frac{2U_1^2}{\hbar v_{\rm F} G_n^{\rm b}}\frac{(\mathbf{e}_z\times\mathbf{K}_{nn})\cdot\boldsymbol{\sigma}}{K_{nn}}\right] e^{-\frac{i}{2}\mathbf{R}\cdot(\mathbf{G}_n^{\rm t}+\mathbf{G}_n^{\rm b})}\, e^{i\mathbf{K}_{nn}\cdot\mathbf{r}}, \end{aligned} \quad (15)$$

where $\mathbf{e}_z$ is the unit vector in the $+z$-direction. As the inversion-symmetry breaking is enhanced by the two aligned B-hBN acting jointly, the gap is twice as big as that induced by a single interface, giving the first term in Equation 15. The effect of both interfaces combined oscillates with the periodicity of the supermoiré lattice, giving the second term in Equation 15.

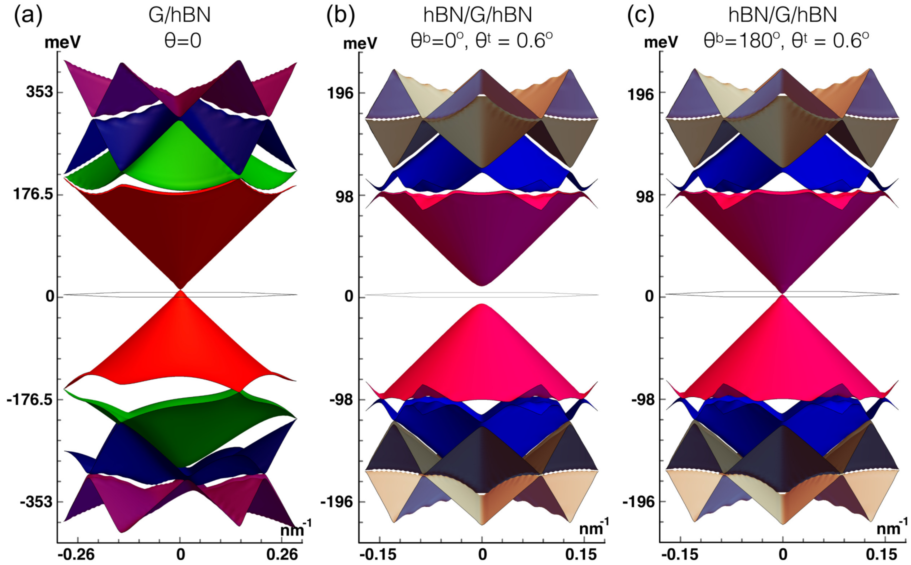

Figure 7: Band structures in the first supermoiré mini BZ for: (a) a single 1L-G/B-hBN interface, (b) H_{mm} parallel alignment, and (c) antiparallel alignment. The dispersion is plotted in the hexagonal BZ.

The perturbative term in the Hamiltonian for $\theta^{\mathrm{t}} = \theta^{\mathrm{b}} + 180^\circ$ is

$$\delta\mathcal{H}_{\mathrm{antipar}} = \sum_{n=1}^{6}\left[i(-1)^m 2U_0 w_{\mathrm{as}} - \frac{4U_1U_3}{\hbar v_{\mathrm{F}} G_n^{\mathrm{b}}} - i\frac{2U_1^2}{\hbar v_{\mathrm{F}} G_n^{\mathrm{b}}}\frac{(\mathbf{e}_z \times \mathbf{K}_{nn})\cdot\boldsymbol{\sigma}}{K_{nn}}\right] e^{-\frac{i}{2}\mathbf{R}\cdot(\mathbf{G}_n^{\mathrm{t}}+\mathbf{G}_n^{\mathrm{b}})}\, e^{i\mathbf{K}_{nn}\cdot\mathbf{r}}. \tag{16}$$

In this case, the large gap (Figure 7b) is canceled by reversing one of the top or bottom B-hBN layers against the other as shown in Figure 7c. Thus, only a small gap due to the inversion-symmetry-breaking components is present. The magnitudes of the different terms are compared in Figure 6b. Figure 7 shows examples of band structures corresponding to the supermoiré Hamiltonians of Equations 15 and 16.

Band-Structure Reconstruction of B-hBN/1L-G/B-hBN: Derivation of the Hamiltonian of Shortest Period

We take into account the combination of the top and bottom single-interface moiré patterns via two mechanisms: (i) quantum-mechanical interference; (ii) lattice reconstruction, where

the total 1L-G strain field is the sum of the strain fields from each interface.

Within second-order perturbation theory, the Hamiltonian for the middle SL generated by the shortest effective reciprocal-lattice vectors $\mathbf{K}_{n,m} = \mathbf{G}^{\rm t}_n - \mathbf{G}^{\rm b}_m$ that combine due to interference is $\mathcal{H}^{\rm int}_{n,m} = \delta\mathcal{H}^{(2)}_{\rm tb} + \delta\mathcal{H}^{(2)}_{\rm bt}$, with

$$
\begin{aligned}
\delta\mathcal{H}^{(2)}_{\substack{\rm tb\\ \rm (bt)}} =& \sum_{n,m} \frac{1}{\hbar v_{\rm F}(G^{\rm b}_m)^2}\Bigg(\Big[U_0U_1(-1)^m\mathbf{G}^{\rm b(t)}_m\cdot\mathbf{e}_m + U_0U_1(-1)^n\mathbf{G}^{\rm b(t)}_m\cdot\mathbf{e}_n \\
&+ U_1U_3(-1)^{n+m}(\mathbf{e}_z\times\mathbf{G}^{\rm b(t)}_m)\cdot(\mathbf{e}_m+\mathbf{e}_n)\Big]e^{\mp i\frac{\mathbf{R}}{2}(\mathbf{G}^{\rm b}_m+\mathbf{G}^{\rm t}_n)}\ e^{i(\mathbf{G}^{\rm b}_m-\mathbf{G}^{\rm t}_n)\cdot\mathbf{r}} \\
&+ i\Big[(-1)^m U_1U_0(\mathbf{e}_z\times\mathbf{G}^{\rm b(t)}_m)\cdot\mathbf{e}_m - (-1)^n U_1U_0(\mathbf{e}_z\times\mathbf{G}^{\rm b(t)}_m)\cdot\mathbf{e}_n \\
&- U_1U_3(-1)^{n+m}\mathbf{G}^{\rm b(t)}_m\cdot(\mathbf{e}_m-\mathbf{e}_n)\Big]\sigma_3\, e^{\mp i\frac{\mathbf{R}}{2}(\mathbf{G}^{\rm b}_m+\mathbf{G}^{\rm t}_n)}\ e^{i(\mathbf{G}^{\rm b}_m-\mathbf{G}^{\rm t}_n)\cdot\mathbf{r}} \\
&+ \Big[i(-1)^{n+m}U_1^2\big[(\mathbf{e}_n\cdot\mathbf{G}^{\rm b(t)}_m)(\mathbf{e}_m\cdot\boldsymbol{\sigma}) + [\mathbf{e}_n\cdot(\mathbf{e}_z\times\mathbf{G}^{\rm b(t)}_m)][(\mathbf{e}_z\times\mathbf{e}_m)\cdot\boldsymbol{\sigma}]\big]\Big]e^{\mp i\frac{\mathbf{R}}{2}\cdot(\mathbf{G}^{\rm b}_m+\mathbf{G}^{\rm t}_n)}\ e^{i(\mathbf{G}^{\rm b}_m-\mathbf{G}^{\rm t}_n)\cdot\mathbf{r}} \\
&+ \Big[U_0^2\mathbf{G}^{\rm b(t)}_m - (-1)^{n+m}U_3^2\mathbf{G}^{\rm b(t)}_m + U_0U_3\big((-1)^m+(-1)^n\big)(\mathbf{e}_z\times\mathbf{G}^{\rm b(t)}_m)\Big]\cdot\boldsymbol{\sigma}e^{\mp i\frac{\mathbf{R}}{2}\cdot(\mathbf{G}^{\rm b}_m+\mathbf{G}^{\rm t}_n)}\ e^{i(\mathbf{G}^{\rm b}_m-\mathbf{G}^{\rm t}_n)\cdot\mathbf{r}}\Bigg).
\end{aligned}
\tag{17}
$$

The terms in the SL Hamiltonian in which combinations $\mathbf{K}_{n,m} = \mathbf{G}^{\rm t}_n - \mathbf{G}^{\rm b}_m$ arise due to lattice reconstruction can be written as

$$
\begin{aligned}
\delta\mathcal{H}^{\rm rec}_{n,m} =& \sum_{n,m}\Bigg(i\Big[U_0^{\rm b}\mathbf{g}_n\cdot\mathbf{u}_{\mathbf{G}^{\rm t}_m} - U_0^{\rm t}\mathbf{g}_n\cdot\mathbf{u}_{-\mathbf{G}^{\rm b}_m}\Big] + i\sigma_3\Big[(-1)^n iU_3^{\rm b}\mathbf{g}_n\cdot\mathbf{u}_{\mathbf{G}^{\rm t}_m} + (-1)^m iU_3^{\rm t}\mathbf{g}_n\cdot\mathbf{u}_{-\mathbf{G}^{\rm b}_m}\Big] \\
&+ \Big[(-1)^n iU_1^{\rm b}\,(\mathbf{e}_n\cdot\boldsymbol{\sigma})\,(\mathbf{g}_n\cdot\mathbf{u}_{\mathbf{G}^{\rm t}_m}) - (-1)^m iU_1^{\rm t}\,(\mathbf{e}_m\cdot\boldsymbol{\sigma})\,(\mathbf{g}_n\cdot\mathbf{u}_{-\mathbf{G}^{\rm b}_m})\Big]\Bigg)\ e^{i(\mathbf{G}^{\rm t}_n+\mathbf{G}^{\rm b}_m)\cdot\frac{\mathbf{R}}{2}}\ e^{i\mathbf{K}_{nm}\cdot\mathbf{r}}.
\end{aligned}
\tag{18}
$$

For each emergent reciprocal-lattice vector of the supermoiré pattern, the corresponding term in the Hamiltonian of Equation 18 is of equivalent form to Equation 13 with $\mathbf{u} = \mathbf{0}$, where the reciprocal-lattice vectors are replaced by those of the supermoiré lattice, and the coefficients are rescaled:

$$
\begin{aligned}
\delta\mathcal{H}^{\rm rec}_{n,m} =& C_{\sigma_0} + \Delta\sigma_3 + \mathbf{A}\cdot\boldsymbol{\sigma} \\
&+ a^{(0)}_{nm}f_1^{(n,m)}(\mathbf{r}) + a^{(3)}_{nm}f_2^{(n,m)}(\mathbf{r})\sigma_3 + a^{(12)}_{nm}[\mathbf{e}_z\times\nabla f_2^{(n,m)}(\mathbf{r})]\cdot\boldsymbol{\sigma} \\
&+ b^{(0)}_{nm}f_2^{(n,m)}(\mathbf{r}) + b^{(3)}_{nm}f_2^{(n,m)}(\mathbf{r})f_1^{(n,m)}(\mathbf{r})\sigma_3 + b^{(12)}_{nm}f_2^{(n,m)}(\mathbf{r})[\mathbf{e}_z\times\nabla f_1^{(n,m)}(\mathbf{r})]\cdot\boldsymbol{\sigma},
\end{aligned}
\tag{19}
$$

where $f_1^{(n,m)}(\mathbf{r}) = \sum_m e^{i(\mathbf{G}_n^{\mathrm{b}}+\mathbf{G}_m^{\mathrm{t}})\cdot\mathbf{R}/2} e^{i\mathbf{K}_{n,m}\cdot\mathbf{r}}$ and $f_2^{(n,m)}(\mathbf{r}) = i\sum_m (-1)^m e^{i(\mathbf{G}_n^{\mathrm{b}}+\mathbf{G}_m^{\mathrm{t}})\cdot\mathbf{R}/2} e^{i\mathbf{K}_{n,m}\cdot\mathbf{r}}$ encode the periodicity of the supermoiré potential. The first line in Equation 19 preserves inversion symmetry, whereas the second line does not. The coupling constants $a_{nm}^{(i)}$ and $b_{nm}^{(i)}$ are given in Tables 3 and 4. The constants in Equation 19 are:

$$g_{\mathrm{al},\perp}^{n,n+1} = -\frac{a_{\mathrm{G}}\left(2\sqrt{3}\delta - 3\theta^{\mathrm{t}} + 3\theta^{\mathrm{b}}\right)}{8\pi\left(\delta^2 + \sqrt{3}\delta(\theta^{\mathrm{b}} - \theta^{\mathrm{t}}) + {\theta^{\mathrm{b}}}^2 - \theta^{\mathrm{b}}\theta^{\mathrm{t}} + {\theta^{\mathrm{t}}}^2\right)}, \tag{20}$$

$$g_{\mathrm{rev},\perp}^{n,n+1} = \frac{3a_{\mathrm{G}}(\theta^{\mathrm{b}} + \theta^{\mathrm{t}})}{8\pi\left(\delta^2 + \sqrt{3}\delta(\theta^{\mathrm{b}} - \theta^{\mathrm{t}}) + {\theta^{\mathrm{b}}}^2 - \theta^{\mathrm{b}}\theta^{\mathrm{t}} + {\theta^{\mathrm{t}}}^2\right)} \tag{21}$$

$$g_{\mathrm{al,s},\perp}^{n,n+2} = g_{\mathrm{rev,as},\perp}^{n,n+2} = \frac{3a_{\mathrm{G}}\left(\sqrt{3}\delta + \theta^{\mathrm{t}} + 2\theta^{\mathrm{b}}\right)}{8\pi\left(3\delta^2 + \sqrt{3}\delta(\theta^{\mathrm{b}} - \theta^{\mathrm{t}}) + {\theta^{\mathrm{b}}}^2 + \theta^{\mathrm{b}}\theta^{\mathrm{t}} + {\theta^{\mathrm{t}}}^2\right)} \tag{22}$$

$$g_{\mathrm{al,as},\perp}^{n,n+2} = g_{\mathrm{rev,s},\perp}^{n,n+2} = -\frac{3a_{\mathrm{G}}\left(2\sqrt{3}\delta + \theta^{\mathrm{b}} - \theta^{\mathrm{t}}\right)}{8\pi\left(3\delta^2 + \sqrt{3}\delta(\theta^{\mathrm{b}} - \theta^{\mathrm{t}}) + {\theta^{\mathrm{b}}}^2 + \theta^{\mathrm{b}}\theta^{\mathrm{t}} + {\theta^{\mathrm{t}}}^2\right)} \tag{23}$$

$$g_{\perp}^{n,n+3} = -\frac{\sqrt{3}a_{\mathrm{G}}}{2\pi}\frac{\delta}{4\delta^2 + (\theta^{\mathrm{b}} + \theta^{\mathrm{t}})^2} \tag{24}$$

$$g_{\mathrm{al,s},\perp}^{n,n+4} = g_{\mathrm{rev,as},\perp}^{n,n+4} = -\frac{3a_{\mathrm{G}}(\theta^{\mathrm{b}} + \theta^{\mathrm{t}})}{8\pi\left(3\delta^2 + \sqrt{3}\delta(\theta^{\mathrm{t}} - \theta^{\mathrm{b}}) + {\theta^{\mathrm{b}}}^2 + \theta^{\mathrm{b}}\theta^{\mathrm{t}} + {\theta^{\mathrm{t}}}^2\right)} \tag{25}$$

$$g_{\mathrm{al,as},\perp}^{n,n+4} = g_{\mathrm{rev,s},\perp}^{n,n+4} - \frac{3a_{\mathrm{G}}\left(2\sqrt{3}\delta + \theta^{\mathrm{t}} - \theta^{\mathrm{b}}\right)}{8\pi\left(3\delta^2 + \sqrt{3}\delta(\theta^{\mathrm{t}} - \theta^{\mathrm{b}}) + {\theta^{\mathrm{b}}}^2 + \theta^{\mathrm{b}}\theta^{\mathrm{t}} + {\theta^{\mathrm{t}}}^2\right)} \tag{26}$$

$$g_{\mathrm{al},\perp}^{n,n+5} = -\frac{a_{\mathrm{G}}\left(2\sqrt{3}\delta - 3\theta^{\mathrm{b}} + 3\theta^{\mathrm{t}}\right)}{8\pi\left(\delta^2 + \sqrt{3}\delta(\theta^{\mathrm{t}} - \theta^{\mathrm{b}}) + {\theta^{\mathrm{b}}}^2 - \theta^{\mathrm{b}}\theta^{\mathrm{t}} + {\theta^{\mathrm{t}}}^2\right)}, \tag{27}$$

$$g_{\mathrm{rev},\perp}^{n,n+5} = \frac{3a_{\mathrm{G}}(\theta^{\mathrm{b}} + \theta^{\mathrm{t}})}{8\pi\left(\delta^2 + \sqrt{3}\delta(\theta^{\mathrm{t}} - \theta^{\mathrm{b}}) + {\theta^{\mathrm{b}}}^2 - \theta^{\mathrm{b}}\theta^{\mathrm{t}} + {\theta^{\mathrm{t}}}^2\right)}. \tag{28}$$

Table 3: Coupling constants for B-hBN/1l-G/B-hBN with aligned B-hBN lattice vectors [$C_{\sigma_0} = -6(U_0^{\mathrm{t}}w_{\mathrm{s}}^{\mathrm{t}} + U_0^{\mathrm{b}}w_{\mathrm{s}}^{\mathrm{b}})$, $\Delta = -6(U_3^{\mathrm{t}}w_{\mathrm{as}}^{\mathrm{t}} + U_3^{\mathrm{b}}w_{\mathrm{as}}^{\mathrm{b}})$, $A_1 = 0$, and $A_2 = 0$]. $w_{\mathrm{as}}^{\mathrm{t/b}} = -(-1)^m\mathrm{Re}(\mathbf{g}_m \cdot \mathbf{u}_{\pm\mathbf{G}_m^{\mathrm{t/b}}})$ and $w_{\mathrm{s}}^{\mathrm{t/b}} = \mathrm{Im}(\mathbf{g}_m \cdot \mathbf{u}_{\pm\mathbf{G}_m^{\mathrm{t/b}}})$

	$a_{nm}^{(0)}$	$a_{nm}^{(3)}$	$a_{nm}^{(12)}$	$b_{nm}^{(0)}$	$b_{nm}^{(3)}$	$b_{nm}^{(12)}$
n, n	$-(U_0^{\mathrm{b}}w_{\mathrm{s}}^{\mathrm{t}} + U_0^{\mathrm{t}}w_{\mathrm{s}}^{\mathrm{b}})$	$-(U_3^{\mathrm{b}}w_{\mathrm{s}}^{\mathrm{t}} - U_3^{\mathrm{t}}w_{\mathrm{s}}^{\mathrm{b}})$	0	$(U_0^{\mathrm{b}}w_{\mathrm{as}}^{\mathrm{t}} - U_0^{\mathrm{t}}w_{\mathrm{as}}^{\mathrm{b}})$	$-(U_3^{\mathrm{b}}w_{\mathrm{as}}^{\mathrm{t}} + U_3^{\mathrm{t}}w_{\mathrm{as}}^{\mathrm{b}})$	0
$n, n+1$	$-\frac{1}{2}(U_0^{\mathrm{b}}w_{\mathrm{s}}^{\mathrm{t}} + U_0^{\mathrm{t}}w_{\mathrm{s}}^{\mathrm{b}})$	$\frac{1}{2}(U_3^{\mathrm{b}}w_{\mathrm{s}}^{\mathrm{t}} - U_3^{\mathrm{t}}w_{\mathrm{s}}^{\mathrm{b}})$	$-\frac{U_1}{2}w_{\mathrm{s}}g_{\mathrm{al},\perp}^{n,n+1}$	$\frac{1}{2}(U_0^{\mathrm{b}}w_{\mathrm{as}}^{\mathrm{t}} + U_0^{\mathrm{t}}w_{\mathrm{as}}^{\mathrm{b}})$	$-\frac{1}{2}(U_3^{\mathrm{b}}w_{\mathrm{as}}^{\mathrm{t}} + U_3^{\mathrm{t}}w_{\mathrm{as}}^{\mathrm{b}})$	$\frac{U_1}{2}w_{\mathrm{as}}g_{\mathrm{al},\perp}^{n,n+1}$
$n, n+2$	$-\frac{1}{2}(U_0^{\mathrm{b}}w_{\mathrm{s}}^{\mathrm{t}} + U_0^{\mathrm{t}}w_{\mathrm{s}}^{\mathrm{b}})$	$-\frac{1}{2}(U_3^{\mathrm{b}}w_{\mathrm{s}}^{\mathrm{t}} - U_3^{\mathrm{t}}w_{\mathrm{s}}^{\mathrm{b}})$	$-\frac{U_1}{2}w_s g_{\mathrm{al,s},\perp}^{n,n+2}$	$\frac{1}{2}(U_0^{\mathrm{b}}w_{\mathrm{as}}^{\mathrm{t}} - U_0^{\mathrm{t}}w_{\mathrm{as}}^{\mathrm{b}})$	$-\frac{U_3}{2}(w_{\mathrm{as}}^{\mathrm{t}} + w_{\mathrm{as}}^{\mathrm{b}})$	$\frac{U_1}{2}w_{\mathrm{as}}g_{\mathrm{al,as},\perp}^{n,n+2}$
$n, n+3$	$(U_0^{\mathrm{b}}w_{\mathrm{s}}^{\mathrm{t}} + U_0^{\mathrm{t}}w_{\mathrm{s}}^{\mathrm{b}})$	$-(U_3^{\mathrm{b}}w_{\mathrm{s}}^{\mathrm{t}} + U_3^{\mathrm{t}}w_{\mathrm{s}}^{\mathrm{b}})$	$-(U_1^{\mathrm{b}}w_{\mathrm{s}}^{\mathrm{t}} + U_1^{\mathrm{t}}w_{\mathrm{s}}^{\mathrm{b}})g_{\perp}^{n,n+3}$	$-(U_0^{\mathrm{b}}w_{\mathrm{s}}^{\mathrm{t}} + U_0^{\mathrm{t}}w_{\mathrm{s}}^{\mathrm{b}})$	$-(U_3^{\mathrm{b}}w_{\mathrm{as}}^{\mathrm{t}} + U_3^{\mathrm{t}}w_{\mathrm{as}}^{\mathrm{b}})$	$(U_1^{\mathrm{b}}w_{\mathrm{as}}^{\mathrm{t}} + U_1^{\mathrm{t}}w_{\mathrm{as}}^{\mathrm{b}})g_{\perp}^{n,n+3}$
$n, n+4$	$-\frac{1}{2}(U_0^{\mathrm{b}}w_{\mathrm{s}}^{\mathrm{t}} + U_0^{\mathrm{t}}w_{\mathrm{s}}^{\mathrm{b}})$	$-\frac{1}{2}(U_3^{\mathrm{b}}w_{\mathrm{s}}^{\mathrm{t}} - U_3^{\mathrm{t}}w_{\mathrm{s}}^{\mathrm{b}})$	$-\frac{U_1}{2}w_{\mathrm{s}}g_{\mathrm{al},\perp}^{n,n+4}$	$\frac{1}{2}(U_0^{\mathrm{b}}w_{\mathrm{as}}^{\mathrm{t}} - U_0^{\mathrm{t}}w_{\mathrm{as}}^{\mathrm{b}})$	$-\frac{1}{2}(U_3^{\mathrm{b}}w_{\mathrm{as}}^{\mathrm{t}} + U_3^{\mathrm{t}}w_{\mathrm{as}}^{\mathrm{b}})$	$\frac{U_1}{2}w_{\mathrm{as}}g_{\mathrm{al,as},\perp}^{n,n+4}$
$n, n+5$	$-\frac{1}{2}(U_0^{\mathrm{b}}w_{\mathrm{s}}^{\mathrm{t}} + U_0^{\mathrm{t}}w_{\mathrm{s}}^{\mathrm{b}})$	$-\frac{1}{2}(U_3^{\mathrm{b}}w_{\mathrm{s}}^{\mathrm{t}} + U_3^{\mathrm{t}}w_{\mathrm{s}}^{\mathrm{b}})$	$-\frac{U_1}{2}w_{\mathrm{s}}g_{\mathrm{al},\perp}^{n,n+5}$	$-\frac{U_0}{2}(w_{\mathrm{as}}^{\mathrm{t}} + w_{\mathrm{as}}^{\mathrm{b}})$	$\frac{1}{2}(U_3^{\mathrm{b}}w_{\mathrm{as}}^{\mathrm{t}} + U_3^{\mathrm{t}}w_{\mathrm{as}}^{\mathrm{b}})$	$-\frac{U_1}{2}w_{\mathrm{as}}g_{\mathrm{al},\perp}^{n,n+5}$

Table 4: Coupling constants for B-hBN/1L-G/B-hBN with anti-aligned B-hBN lattice vectors [$C_{\sigma_0} = -6(U_0^{\rm t} w_{\rm s}^{\rm t} - U_0^{\rm b} w_{\rm s}^{\rm b})$, $\Delta = -6(U_3^{\rm t} w_{\rm as}^{\rm t} - U_3^{\rm b} w_{\rm as}^{\rm b})$, $A_1 = 0$, and $A_2 = 0$].

	$a_{nm}^{(0)}$	$a_{nm}^{(3)}$	$a_{nm}^{(12)}$	$b_{nm}^{(0)}$	$b_{nm}^{(3)}$	$b_{nm}^{(12)}$
n, n	$-(U_0^{\rm b} w_{\rm s}^{\rm t} - U_0^{\rm t} w_{\rm s}^{\rm b})$	$-(U_3^{\rm b} w_{\rm s}^{\rm t} + U_3^{\rm t} w_{\rm s}^{\rm b})$	0	$U_0(w_{\rm as}^{\rm t} + w_{\rm as}^{\rm b})$	$-(U_3^{\rm b} w_{\rm as}^{\rm t} - U_3^{\rm t} w_{\rm as}^{\rm b})$	0
$n, n+1$	$-\frac{1}{2}(U_0^{\rm b} w_{\rm s}^{\rm t} - U_0^{\rm t} w_{\rm s}^{\rm b})$	$\frac{1}{2}(U_3^{\rm b} w_{\rm s}^{\rm t} + U_3^{\rm t} w_{\rm s}^{\rm b})$	$-\frac{U_1}{2} w_{\rm s} g_{{\rm rev},\perp}^{n,n+1}$	$\frac{1}{2}(U_0^{\rm b} w_{\rm as}^{\rm t} - U_0^{\rm t} w_{\rm as}^{\rm b})$	$-\frac{1}{2}(U_3^{\rm b} w_{\rm as}^{\rm t} - U_3^{\rm t} w_{\rm as}^{\rm b})$	$\frac{U_1}{2} w_{\rm as} g_{{\rm rev},\perp}^{n,n+1}$
$n, n+2$	$-\frac{1}{2}(U_0^{\rm b} w_{\rm s}^{\rm t} - U_0^{\rm t} w_{\rm s}^{\rm b})$	$-\frac{1}{2}(U_3^{\rm b} w_{\rm s}^{\rm t} + U_3^{\rm t} w_{\rm s}^{\rm b})$	$-\frac{U_1}{2} w_s g_{{\rm rev,s},\perp}^{n,n+2}$	$\frac{1}{2}(U_0^{\rm b} w_{\rm as}^{\rm t} + U_0^{\rm t} w_{\rm as}^{\rm b})$	$-\frac{U_3}{2}(w_{\rm as}^{\rm t} - w_{\rm as}^{\rm b})$	$\frac{U_1}{2} w_{\rm as} g_{{\rm rev,as},\perp}^{n,n+2}$
$n, n+3$	$(U_0^{\rm b} w_{\rm s}^{\rm t} - U_0^{\rm t} w_{\rm s}^{\rm b})$	$-(U_3^{\rm b} w_{\rm s}^{\rm t} - U_3^{\rm t} w_{\rm s}^{\rm b})$	$-(U_1^{\rm b} w_{\rm s}^{\rm t} - U_1^{\rm t} w_{\rm s}^{\rm b}) g_\perp^{n,n+3}$	$-(U_0^{\rm b} w_{\rm s}^{\rm t} - U_0^{\rm t} w_{\rm s}^{\rm b})$	$-(U_3^{\rm b} w_{\rm as}^{\rm t} - U_3^{\rm t} w_{\rm as}^{\rm b})$	$(U_1^{\rm b} w_{\rm as}^{\rm t} - U_1^{\rm t} w_{\rm as}^{\rm b}) g_\perp^{n,n+3}$
$n, n+4$	$-\frac{1}{2}(U_0^{\rm b} w_{\rm s}^{\rm t} - U_0^{\rm t} w_{\rm s}^{\rm b})$	$-\frac{1}{2}(U_3^{\rm b} w_{\rm s}^{\rm t} + U_3^{\rm t} w_{\rm s}^{\rm b})$	$-\frac{U_1}{2} w_{\rm s} g_{{\rm rev},\perp}^{n,n+4}$	$\frac{1}{2}(U_0^{\rm b} w_{\rm as}^{\rm t} + U_0^{\rm t} w_{\rm as}^{\rm b})$	$-\frac{1}{2}(U_3^{\rm b} w_{\rm as}^{\rm t} - U_3^{\rm t} w_{\rm as}^{\rm b})$	$\frac{U_1}{2} w_{\rm as} g_{{\rm rev,as},\perp}^{n,n+4}$
$n, n+5$	$-\frac{1}{2}(U_0^{\rm b} w_{\rm s}^{\rm t} - U_0^{\rm t} w_{\rm s}^{\rm b})$	$-\frac{1}{2}(U_3^{\rm b} w_{\rm s}^{\rm t} - U_3^{\rm t} w_{\rm s}^{\rm b})$	$-\frac{U_1}{2} w_{\rm s} g_{{\rm rev},\perp}^{n,n+5}$	$-\frac{U_0}{2}(w_{\rm as}^{\rm t} - w_{\rm as}^{\rm b})$	$\frac{1}{2}(U_3^{\rm b} w_{\rm as}^{\rm t} - U_3^{\rm t} w_{\rm as}^{\rm b})$	$-\frac{U_1}{2} w_{\rm as} g_{{\rm rev},\perp}^{n,n+5}$

Time-Step Errors in DMC Calculations

To investigate the finite-time-step errors in our DMC energies, we calculated the non-twist-averaged ground-state DMC energy of 1L-G and 1L-hBN as well as the BE of 1L-G/1L-hBN (stacking configuration II) for a supercell composed of 3×3 primitive cells. Figure 8 shows that the time-step errors in the total energies at a time step of 0.04 Ha^{-1} are typically ~ 30 meV/atom, but these errors partially cancel when the BE is calculated. The DMC total energy of each 1L varies linearly with time step, up to ~ 0.04 Ha^{-1}. The nonlinear contribution to the time-step bias is primarily due to the atomic cores, and hence largely cancels out of the BE, which exhibits linear time-step bias up to a much larger time step of ~ 0.2 Ha^{-1}. We therefore extrapolate all our final DMC BEs to zero time step using time steps of 0.04 and 0.1 Ha^{-1}.

References

(1) Efron, B.; Tibshirani, R. J. *An Introduction to the Bootstrap*; Chapman and Hall/CRC, 1994.

(2) Mostaani, E.; Drummond, N. D.; Fal'ko, V. I. Quantum Monte Carlo Calculation of the Binding Energy of Bilayer Graphene. *Phys. Rev. Lett.* **2015**, *115*, 115501.

(3) Giovannetti, G.; Khomyakov, P. A.; Brocks, G.; Kelly, P. J.; van den Brink, J.

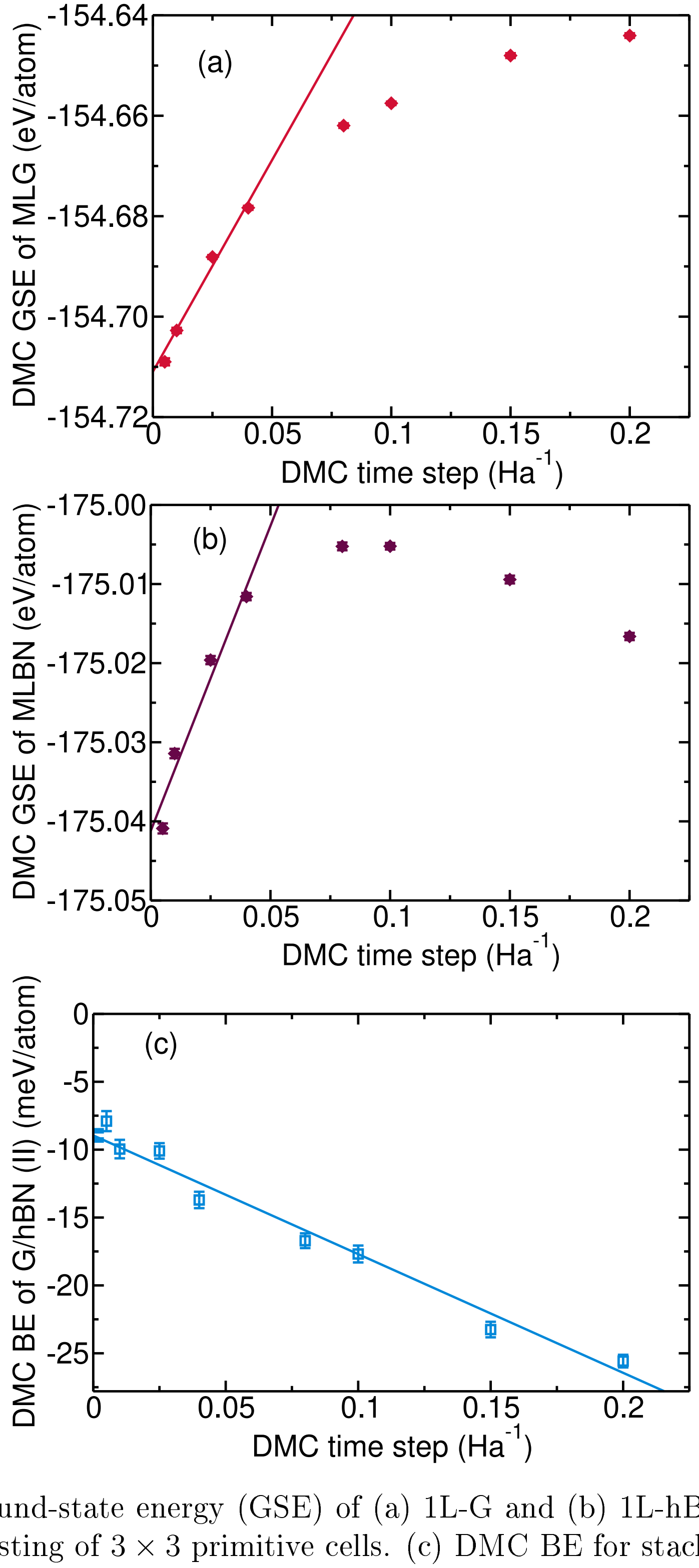

Figure 8: DMC ground-state energy (GSE) of (a) 1L-G and (b) 1L-hBN against time step for a supercell consisting of 3×3 primitive cells. (c) DMC BE for stacking configuration II of 1L-G/1L-hBN against time step. These DMC results are not twist averaged.

Substrate-Induced Band Gap in Graphene on Hexagonal Boron Nitride: *Ab Initio* Density Functional Calculations. *Phys. Rev. B* **2007**, *76*, 073103.

(4) Fan, Y.; Zhao, M.; Wang, Z.; Zhang, X.; Zhang, H. Tunable Electronic Structures of Graphene/Boron Nitride Heterobilayers. *Appl. Phys. Lett.* **2011**, *98*, 083103.

(5) Sachs, B.; Wehling, T. O.; Katsnelson, M. I.; Lichtenstein, A. I. Adhesion and Electronic Structure of Graphene on Hexagonal Boron Nitride Substrates. *Phys. Rev. B* **2011**, *84*, 195414.

(6) Slotman, G. J.; de Wijs, G. A.; Fasolino, A.; Katsnelson, M. I. Phonons and Electron-Phonon Coupling in Graphene-h-BN Heterostructures. *Ann. Phys. (Berl.)* **2014**, *526*, 381–386.

(7) Leconte, N.; Jung, J.; Lebègue, S.; Gould, T. Moiré-Pattern Interlayer Potentials in van der Waals Materials in the Random-Phase Approximation. *Phys. Rev. B* **2017**, *96*, 195431.

(8) Feynman, R. P. Forces in Molecules. *Phys. Rev.* **1939**, *56*, 340–343.

(9) Blakslee, O. L.; Proctor, D. G.; Seldin, E. J.; Spence, G. B.; Weng, T. Elastic Constants of Compression-Annealed Pyrolytic Graphite. *J. Appl. Phys.* **1970**, *41*, 3373–3382.

(10) Fayos, J. Possible 3D Carbon Structures as Progressive Intermediates in Graphite to Diamond Phase Transition. *J. Solid State Chem.* **1999**, *148*, 278–285.

(11) Lee, C.; Wei, X.; Kysar, J. W.; Hone, J. Measurement of the Elastic Properties and Intrinsic Strength of Monolayer Graphene. *Science* **2008**, *321*, 385–388.

(12) Falin, A.; Cai, Q.; Santos, E. J. G.; Scullion, D.; Qian, D.; Zhang, R.; Yang, Z.; Huang, S.; Watanabe, K.; Taniguchi, T.; Barnett, M. R.; Chen, Y.; Ruoff, R. S.; Li, L. H. Mechanical Properties of Atomically Thin Boron Nitride and the Role of Interlayer Interactions. *Nat. Commun.* **2017**, *8*, 15815.

(13) San-Jose, P.; Gutiérrez-Rubio, A.; Sturla, M.; Guinea, F. Spontaneous Strains and Gap in Graphene on Boron Nitride. *Phys. Rev. B* **2014**, *90*, 075428.

(14) Hestenes, M. R.; Stiefel, E. Methods of Conjugate Gradients for Solving Linear Systems. *J. Res. Natl. Bur. Stand.* **1952**, *49*, 409–435.

(15) Wang, L.; Zihlmann, S.; Liu, M.-H.; Makk, P.; Watanabe, K.; Taniguchi, T.; Baumgartner, A.; Schönenberger, C. New Generation of Moiré Superlattices in Doubly Aligned hBN/Graphene/hBN Heterostructures. *Nano Lett.* **2019**, *19*, 2371–2376.

(16) Wang, Z. et al. Composite Super-Moiré Lattices in Double-Aligned Graphene Heterostructures. *Sci. Adv.* **2019**, *5*, eaay8897.

(17) Finney, N. R.; Yankowitz, M.; Muraleetharan, L.; Watanabe, K.; Taniguchi, T.; Dean, C. R.; Hone, J. Tunable Crystal Symmetry in Graphene-Boron Nitride Heterostructures with Coexisting Moiré Superlattices. *Nat. Nanotechnol.* **2019**, *14*, 1029–1034.

(18) Yang, Y.; Li, J.; Yin, J.; Xu, S.; Mullan, C.; Taniguchi, T.; Watanabe, K.; Geim, A. K.; Novoselov, K. S.; Mishchenko, A. *In Situ* Manipulation of van der Waals Heterostructures for Twistronics. *Sci. Adv.* **2020**, *6*, eabd3655.

(19) Anđelković, M.; Milovanović, S. P.; Covaci, L.; Peeters, F. M. Double Moiré with a Twist: Supermoiré in Encapsulated Graphene. *Nano Lett.* **2020**, *20*, 979–988.

(20) Sun, X. et al. Correlated States in Doubly-Aligned hBN/Graphene/hBN Heterostructures. *Nat. Commun.* **2021**, *12*, 7196.

(21) Moulsdale, C.; Knothe, A.; Fal'ko, V. Kagome Network of Miniband-Edge States in Double-Aligned Graphene-Hexagonal Boron Nitride Structures. *Phys. Rev. B* **2022**, *105*, L201112.

(22) Hu, J.; Tan, J.; Al Ezzi, M. M.; Chattopadhyay, U.; Gou, J.; Zheng, Y.; Wang, Z.; Chen, J.; Thottathil, R.; Luo, J.; Watanabe, K.; Taniguchi, T.; Wee, A. T. S.; Adam, S.; Ariando, A. Controlled Alignment of Supermoiré Lattice in Double-Aligned Graphene Heterostructures. *Nat. Commun.* **2023**, *14*, 4142.

(23) Wallbank, J. R.; Patel, A. A.; Mucha-Kruczyński, M.; Geim, A. K.; Fal'ko, V. I. Generic Miniband Structure of Graphene on a Hexagonal Substrate. *Phys. Rev. B* **2013**, *87*, 245408.

(24) Novoselov, K. S.; Geim, A. K.; Morozov, S. V.; Jiang, D.; Katsnelson, M. I.; Grigorieva, I. V.; Dubonos, S. V.; Firsov, A. A. Two-Dimensional Gas of Massless Dirac Fermions in Graphene. *Nature* **2005**, *438*, 197–200.

(25) Cosma, D. A.; Wallbank, J. R.; Cheianov, V.; Fal'ko, V. I. Moiré Pattern as a Magnifying Glass for Strain and Dislocations in van der Waals Heterostructures. *Faraday Discuss.* **2014**, *173*, 137–143.
